# Colorectal cancer risk prediction using a simple multivariable model

**DOI:** 10.1371/journal.pone.0321641

**Published:** 2025-05-13

**Authors:** Gillian S. Dite, Chi Kuen Wong, Aviv Gafni, Erika Spaeth

**Affiliations:** 1 Genetic Technologies Limited, Fitzroy, Victoria, Australia; 2 geneType Inc, Charlotte, North Carolina, United States of America; University of Auckland, NEW ZEALAND

## Abstract

Accurate population stratification of colorectal cancer risk enables identification of individuals who would benefit from screening and risk-reducing interventions. We conducted a population-based cohort study using almost 400,000 unaffected UK Biobank participants who were aged 40–69 years at their baseline assessment and who had genetically determined UK ancestry. For women and men separately, we developed (i) a multivariable risk prediction model using family history, a polygenic risk score (PRS) and clinical risk factors, and (ii) a simple model comprising family history and a PRS. We then compared their performance to that of existing models. The models were developed using Cox regression with age as the time axis in a 70% training dataset. The performance of the 10-year risk of colorectal cancer was assessed in a 30% testing dataset using Cox regression to estimate the hazard ratio per standard deviation of risk, Harrell’s C-index to assess discrimination and logistic regression to assess calibration. There were 214,183 women and 181,889 men in the dataset with 1,913 women and 2,598 men diagnosed with colorectal cancer during the follow-up period. The mean age at diagnosis was 66.4 years (standard deviation = 7.3 years) for women and 67.3 years (standard deviation = 6.7 years) for men. In the 30% testing dataset, the new multivariable models discriminated better (Harrell’s C-index = 0.690, 95% CI = 0.669 to 0.712 for women; 0.699, 95% CI = 0.681 to 0.717 for men) than the new family history and PRS models (Harrell’s C-index = 0.683, 95% CI = 0.663 to 0.704 for women; 0.692, 95% CI = 0.673 to 0.710 for men; change in discrimination P = 0.02 for women and P = 0.01 for men). Our models identify individuals who are at increased risk of colorectal cancer and who would benefit from personalised screening and risk-reduction options.

## Introduction

Current population-based screening for colorectal cancer is based on age, with the age of screening initiation ranging from 45 to 60 years in developed countries [1]. The mode of screening is most commonly a faecal immunochemical test every one or two years, but a notable exception is the United States, where colonoscopy every 10 years is recommended [2,3]. Additional screening (either earlier, more frequent or a different mode) is offered to people who are thought to be at increased risk because they have inflammatory bowel disease, a family history of colorectal cancer or a high-penetrance variant in a colorectal cancer predisposition gene [3–6]. But this might not be the best approach because only 10% of the population have an affected first-degree relative or a high-penetrance variant [7]; 70% of colorectal cancers occur in people not known to be at increased risk [8].

There is a need to improve the identification of people at high risk of colorectal cancer so that additional screening can be better targeted to those who need it most. A precision medicine approach to colorectal cancer screening – where a risk prediction model that uses family history of colorectal cancer with additional risk factors is used to stratify the population along the continuum of risk – could help determine recommendations for initiation, interval and mode of screening for each person based on their predicted risk. Taking advantage of the cost-effectiveness [9] and mortality benefit [10] of current colorectal cancer screening options, a precision medicine approach would lead to improved screening compliance, earlier detection and prevention opportunities in higher risk individuals that would reflect in healthcare cost-savings [11–13].

Over the years, many colorectal cancer risk prediction models have been developed of varying complexity and varying predictive performance [14,15]. Colorectal cancer is more common in men than in women [16], and the risk prediction models often perform better for men than for women [17–19]. Polygenic risk scores (PRSs) summarise the cumulative effects of low-penetrance single-nucleotide polymorphisms (SNPs) that are identified from genome-wide association studies as having a small but statistically significant effect on disease risk [20,21]. These PRSs have been used to improve clinical risk prediction for many diseases [22], and we and others have shown that the addition of a PRS can improve colorectal cancer risk prediction [23–25].

In busy primary healthcare settings, simple models with few easy-to-collect risk factors are easier to implement than models with risk factors that are numerous, complicated or not readily available. Therefore, if two models have similar performance, the simple model might be preferable for implementation. In this study we aimed to develop new 10-year risk prediction models for colorectal cancer – separately for women and men – using (i) a 140-SNP PRS [26], first-degree family history and simple and readily available clinical risk factors and (ii) a simple model comprising the 140-SNP PRS and first-degree family history. We then compared the performance of the risk predictions of the new models to (i) average risks based on population incidence rates, and risk predictions from our previous model [23] using (ii) first-degree family history alone and (iii) first-degree family history and a 140-SNP PRS [25] (instead of the original 45-SNP PRS).

## Methods

### UK Biobank

From 2006 to 2009, the UK Biobank recruited over 500,000 participants from England, Wales and Scotland [27–29]. The in-person baseline assessment included completing a touch-screen questionnaire and a face-to-face interview with a nurse. Participants also had physical measurements taken and provided samples of blood, urine and saliva, from which comprehensive genomic and biomarker data has been made available. Additional data has been obtained through linkage to cancer registries, death registries and hospital records, and around 45% have linked primary care data available for analysis [30]. The UK Biobank’s 5.5% participation rate has resulted in a cohort that is not representative of population of the United Kingdom [31]. But, despite the presence of a healthy volunteer selection bias, the results of analyses of exposures and diseases are generalisable because of the vast size of the resource and the substantial variation in exposure measures [31].

### Eligibility

We extracted data for participants who had not withdrawn their consent by 25 April 2023, whose genetic sex was the same as their gender identity and who were aged 40–60 years at their baseline assessment. Participants were excluded from this study if they had been diagnosed with colorectal cancer before their baseline assessment date; did not have genotyping data available; had a history of polyps, Chron’s disease or ulcerative colitis; or had been diagnosed with colorectal cancer or died within the first six weeks of follow-up. So that the dataset did not have closely related pairs of participants, we used the ukb_gen_samples_to_remove function of the R package ukbtools [32,33]. This function identifies related pairs (we used a criterion of closer than third-degree relatedness) and randomly chooses one to be removed from the dataset. The last step was to restrict the dataset to participants who had a genetically determined UK ancestry. Table 1 provides details of the eligibility criteria and the number of participants eligible and dropped at each step.

**Table 1:** Eligibility criteria and the number eligible and dropped at each step.

N eligible	Criteria	N dropped
502,366	Active UK Biobank participant (on 25 April 2023)	
501,988	Gender identity same as genetic sex	378
498,841	Aged 40–69 years at baseline assessment date	3,147
495,977	No colorectal cancer at baseline assessment date	2,864
480,978	Genotyping data available	14,999
466,459	No history of polyps, Chron’s disease or ulcerative colitis	14,519
466,426	Alive after six weeks of follow-up	33
466,399	Unaffected after six weeks of follow-up	27
434,476	Unrelated individuals (≥3rd degree relatedness)	31,923
396,072	Genetically determined UK ancestry	38,404

### Data extraction

Details of the UK Biobank data fields used to derive variables for analysis and eligibility assessment are in S1 Table. For first-degree family history of colorectal cancer, the data fields cover mother, father and any sibling; there is no way of knowing if more than one sibling has been affected. Very few participants (0.5%) had two or more affected first-degree relatives; therefore, we used family history as a binary variable for having any affected first-degree relative. The physical activity fields were combined into a summary measure, using the short format calculation of the metabolic equivalent of task in Craig et al [34] and then dividing by 1,000. For women whose menopausal status was unknown (because of hysterectomy or another reason), their status was adjudicated using hormone replacement therapy (HRT) use (menopausal if she had ever taken HRT) and age at baseline assessment (for women who had never taken HRT, premenopausal if aged <51 years and menopausal if aged ≥51 years). Menopause and HRT use were combined into a single risk factor with categories for premenopausal, menopausal with no HRT and menopausal with HRT. To identify participants with genetically determined United Kingdom ancestry, we used the ancestry categories that were defined by principal components analysis by Privé et al [35] and made available for download from the UK Biobank.

We extracted genotypes for the panel of 140 SNPs [26] from the UK Biobank’s SNP imputation dataset using Plink version 1.9 [36,37]. In the published list of SNPs (S4 Table in their supplementary material [26]), the rsID for 13:34092164_C/T had been mis-identified as rs377429877 and should have been rs9537756. Overall, 58,124 (14.7%) participants had all 140 SNPs genotyped, 110,695 (28.0%) were missing one SNP and 106,068 (26.8%) were missing two SNPs. Only 18,888 (4.8%) were missing five or more SNPs. The PRS for use in developing the new models was calculated as the linear combination of the published betas [26] multiplied by the number of effect alleles for each SNP and then standardised to have a mean of 0 and a standard deviation (SD) of 1. We also calculated a population-adjusted PRS [38] (as a relative risk) for use in the current family history and PRS model.

### Statistical analysis

For each participant, follow-up began at the date of their baseline assessment and ended at the earliest of their date of diagnosis of colorectal cancer or 31 July 2019 (the date to which linkage to cancer registries was complete). The participants were randomly divided into a 70% training dataset and a 30% testing dataset that were balanced for sex and affected status.

In the training dataset, we used all available follow-up, while in the testing dataset, we limited follow-up to 10 years. For the calculation of standardised incidence ratios (SIR) in the testing dataset, follow-up was censored at age of death for participants who had died before completing 10 years of follow-up. Stata (version 18.0) [39] was used for most of the analyses; R [33] was used for the variable selection for the new multivariable models for men and women. All statistical tests were two sided and P values < 0.05 were considered nominally statistically significant.

#### Training.

Some of the risk factors considered for inclusion in the models had missing data, most notably physical activity, which was missing for 25.2% unaffected and 27.8% affected women and for 17.6% unaffected and 19.4% affected men (S2 Table). The lipid profile measures were missing for 4.7–13.4% unaffected and 4.7–12.9% affected women and 4.6–11.9% unaffected and 5.3–12.4% affected men. Other risk factors had missing data for less than 2.0% of the participants. We therefore used multiple imputation in the training dataset.

After a pilot analysis using 11 imputations, we determined the required number of imputations using von Hippel’s two-stage approach [40,41]. This calculation uses the upper limit of the 95% confidence interval for the fraction of missing information as input (rather than the point estimate) to ensure that there is only a 2.5% chance that the required number of imputations will be underestimated. With all variables under consideration included, 30 imputations were required, a number driven by the large proportion of missing information for physical activity and the blood lipid measures (omitting physical activity reduced the number of imputations needed to 7; also omitting the blood lipids reduced the number to two).

Given the lengthy computation time required for each imputation, we took a pragmatic approach and repeated the calculation for all variables using the point estimate of the fraction of missing information (0.25) and used 14 imputations for development of the models. Once the models were developed, we repeated the calculation using the upper limit of the 95% confidence interval for the fraction of missing information as the input to ensure that the number of imputations was adequate.

For the imputations, we used chained equations: linear regression for body mass index, physical activity and the four lipid profile measures; logistic regression for first-degree family history, smoking ever, NSAID use, vitamin D supplements, calcium supplements, fish oil supplements/oily fish intake and dried fruit intake; truncated regression (with an allowed range from 0 to 10) for fresh fruit intake, cooked vegetable intake and raw vegetable/salad intake; predictive mean matching (with 3 nearest neighbours) for cereal intake, wholemeal/wholegrain bread intake and white bread intake; conditional multinomial logistic regression for combined menopause and HRT status for women only; and ordered logistic regression for alcohol use, colorectal cancer screening; processed meat intake, beef intake and pork intake.

Using the multiple imputation training dataset, we used age (in years) as the time axis and fitted Cox proportional hazards models for women and men separately. We first obtained unadjusted hazard ratios for each of the risk factors considered for inclusion in the models. For the new multivariable models, we performed forwards and backwards stepwise model selection separately on each of the 14 imputed datasets, and for women and men separately. To obtain simple models, we used the Bayesian information criterion as the measure of performance because it penalises additional parameters more than the Akaike information criterion. After the stepwise procedures, we selected variables that appeared in at least half of the models. We fitted Cox proportional hazards models for women and men separately using the selected variables and used Wald tests to determine whether the variables would be retained in the final models. As an alternative to the new multivariable models, we also fitted new models for women and men with only PRS and first-degree family history as covariates.

We tested the proportional hazards assumption of the new models by including each of the variables as a time-varying covariate. Because this test is sensitive to small deviations from the assumption, we assessed any potentially problematic variables using a plot of the scaled Schoenfeld residuals by age in the first imputation dataset. The fit of the models was assessed using a graph of the Nelson–Aalen cumulative hazard function and the Cox–Snell residuals for the first imputation dataset.

To directly compare the strength of the associations for each of the risk factors (which had been measured on different scales) in the new models, we used the odds per adjusted SD approach [42]. In all 14 of the imputation datasets, we used each risk factor as the dependent variable and fitted a linear or logistic regression (as appropriate) with the other risk factors as independent variables. We then obtained the residuals from these models and divided these by their SD. These new variables were then included in Cox regression models. The estimates and standard errors from the 14 imputation datasets for each of the new models were combined using Rubin’s rules [43] before calculation of the 95% confidence intervals and P values.

#### Testing.

The risk factors included in the new multivariable models for women and men had little missing data in the testing dataset: under 0.5% for women and under 1.4% for men (except for triglycerides, which was missing for 4.8% of women). We therefore replaced missing values with the reference value for the categorical variables and the mean value for the continuous variables. For the new models, we calculated the linear combination of the risk factors and beta coefficients for each participant and centred this value by subtracting the mean. We then obtained the natural exponential of these centred values and used these as the relative risk in the calculation of absolute risk.

For the current family history and PRS model, we multiplied the population-adjusted PRS by 0.92 if the participant had no first-degree family history of colorectal cancer and by 2.10 if they did, as in Gafni et al [23]. For the family history alone model, we assigned a value of 0.92 if the participant had no first-degree family history of colorectal cancer and 2.10 if they did (to ensure that the population average risk was equal to 1). We then used these values as the relative risk in the calculation of the absolute risks. Population average risks were calculated using the equations below without a relative risk term.

For the calculation of absolute 10-year risks of colorectal cancer in the testing dataset, we used annual, sex-specific, age-specific and age-standardised population incidences for England [44]. For the 10-year risks we applied the competing mortality adjustment in equation 5 of Gail et al [45] using annual sex- and age-specific non-colorectal cancer mortality rates from England and Wales [46,47]. These incidences and mortality rates are annual and constant in 5- or 10-year periods, so, as in equation 6 of Gail et al [45], they reduce to the following explicit formulae.

Let $\lambda_{1}(t)$ be the relative risk multiplied by the population colorectal cancer incidence for a woman aged t years. Let $\lambda_{2}(t)$ be the non-colorectal cancer mortality rates for an individual aged t years. Assume that $\lambda_{1}(t)$ is a step function of t that is constant for t in all intervals of the form [k,  k+1) for an integer k (this holds true for our incidences and rates, in fact they are constant in larger, 5- or 10-year, intervals). This is the same assumption as in Gail et al [45] with ${\;\tau }_{j}=j+1$ and ${\;\Delta }_{j}=1$, in their terminology. Then, equation 6 of Gail et al [45] says that the probability that an individual will develop colorectal cancer in the next 10 years, given that the individual is currently unaffected and aged *a* years, is the 10-year risk


$$\sum_{j=a}^{a+9}\frac{\lambda_{1}(j)}{\lambda_{1}(j)+\lambda_{2}(j)}\frac{{\;S}_{1}(j)}{{\;S}_{1}(a)}\frac{{\;S}_{2}(j)}{{\;S}_{2}(a)}[1-exp(-\lambda_{1}(j)-\lambda_{2}(j))],$$

where


$$S_{1}(t)=exp(-\lambda_{1}(0)-\lambda_{1}(1)-\lambda_{1}(2)-\ldots -\lambda_{1}(t-1))$$

if t≥1 is an integer, and $S_{2}(t)$ has the same definition except with a subscript of 2 instead of 1. We calculated the full lifetime colorectal cancer risks using the equations above for ages j=0 to j=89.

We conducted analyses of the performance of the 10-year risk predictions in the 30% testing dataset for women and men separately for the: average risk model, family history alone model, current family history and PRS model, new family history and PRS model, and new multivariable model. We used Cox regression with age as the time axis to estimate the hazard ratio (HR) per SD of the log odds of the 10-year risks. We used Harrell’s C-index to assess the ability of the risk predictions to distinguish between affected and unaffected participants (i.e., the discrimination of the risk scores). Comparisons of the Harrell’s C-index were conducted using DeLong’s [48] method if the indices were correlated and using Zhou’s [49] method if the indices were independent.

We evaluated calibration using logistic regression to estimate coefficients for the log odds of the predicted 10-year risk for the risk scores and tested whether the coefficients were equal to 1 [50,51]. The estimated coefficient is a measure of dispersion, where values < 1 indicate over-dispersion, values > 1 indicate under-dispersion and values close to 1 indicate no problem with dispersion. We then constrained the logistic regression models to have a slope of 1 and used the intercept term to assess overall calibration [51]. To illustrate the calibration of the models, we drew calibration plots for deciles of the 10-year risks using the pmcalplot module [52] in Stata [39].

We then plotted Nelson–Aalen cumulative hazard curves for the risk scores stratified by quintile of 10-year risk and extracted the cumulative hazards at ages 55, 65 and 75 years. We also analysed the performance of the models stratified by 10-year age.

We assessed the extent to which the testing dataset represented women and men in the United Kingdom population by estimating the standardised incidence ratio (SIR) of the number of colorectal cancers expected using sex-specific, age-specific and calendar year-specific population incidence rates for England [44] compared to the number observed during the 10 years of follow-up, overall and by 10-year age group. To illustrate the ability of the models to stratify colorectal cancer risk, we then calculated the SIR of the number of cases expected using sex- and age-specific population incidence rates for England [44] and the number observed during the 10 years of follow-up, overall and for the first four quintiles and the top two deciles of 10-year risk.

### Ethics approval

The UK Biobank has Research Tissue Bank approval (REC #11/NW/0382) that covers analysis of data by approved researchers. All participants provided written informed consent to the UK Biobank before data collection began. This research has been conducted using the UK Biobank resource under Application Number 47401.

## Results

After exclusions, there were 396,072 participants (214,183 women and 181,889 men) in the study dataset, 4,511 (1,913 women and 2,598 men) of whom were diagnosed with incident colorectal cancer during the follow-up period. Mean age at baseline assessment date was 57.0 years (SD = 7.8 years) for unaffected women, 60.7 years (SD = 6.7) for affected women, 57.3 years (SD = 8.0 years) for unaffected men and 61.7 (SD = 6.2 years) for affected men. For affected participants, the mean age at diagnosis was 66.4 years (SD = 7.3 years) for women and 67.3 years (SD = 6.7 years) for men. Affected participants had a mean follow-up time until their diagnosis of 5.7 years (SD = 3.0 years) for women and 5.6 years (SD = 3.0 years) for men. Unaffected participants had a mean follow-up time of 10.4 years (SD = 1.2 years) for women and 10.2 years (SD = 1.5 years) for men. Summary statistics for unaffected and affected women and men for the baseline risk factors considered in the development of the colorectal cancer risk prediction models are presented in S2 Table.

### Training

The unadjusted hazard ratios for the 70% training dataset are shown in S3 Table and the new multivariable models for women and men are presented in Table 2. For both women and men, using forwards selection and backwards selection gave the same set of variables. PRS, first-degree family history of colorectal cancer, smoke ever and colorectal cancer screening were selected for the models for both women and men; triglycerides was selected for the model for women, whereas body mass index was selected for men. All selected variables were statistically significant based on Wald tests, and all were retained in the final models. The number of imputations required to ensure that the standard errors are replicable were six for the model for women and two for the model for men (the upper limits of the 95% confidence interval for the fraction of missing information were 0.15 and 0.04, respectively).

**Table 2:** Hazard ratios for the risk factors in the new models for women and men in the 70% training dataset.

Risk factor	Hazard ratio	95% confidence interval	P value
**Women – new multivariable model**			
140-SNP PRS (standardised)	1.515	1.434, 1.601	<0.001
Affected first-degree relative, any	1.238	1.061, 1.444	0.007
Smoking, ever	1.242	1.115, 1.323	<0.001
Screening procedure in last 10 years, yes	0.594	0.471, 0.749	<0.001
Triglycerides (mmol/L, centred)	1.100	1.038, 1.166	0.001
**Women – new family history and PRS model**			
140-SNP PRS (standardised)	1.514	1.433, 1.599	<0.001
Affected first-degree relative, any	1.212	1.039, 1.413	0.02
**Men – new multivariable model**			
140-SNP PRS (standardised)	1.492	1.423, 1.564	<0.001
Affected first-degree relative, any	1.387	1.226, 1.570	<0.001
Smoking, ever	1.343	1.220, 1.478	<0.001
Screening procedure in last 10 years, yes	0.669	0.540, 0.828	<0.001
Body mass index (natural log of kg/m^2^, centred)	2.410	1.760, 3.301	<0.001
**Men – new family history and PRS model**			
140-SNP PRS (standardised)	1.493	1.424, 1.565	<0.001
Affected first-degree relative, any	1.375	1.214, 1.558	<0.001

Note: PRS, polygenic risk score.

The new family history and PRS models for both women and men are also shown in Table 2. For both women and men, the hazard ratios for the PRS and first-degree family history were similar in the two models. S1 Fig shows the graphs of the Nelson–Aalen cumulative hazard function and the Cox–Snell residuals for the first imputation dataset for each of the new models. In each case, the models were good fits to the data. For women, fitting the risk factors as time-varying covariates did not identify any problems with the proportional hazards assumption in both of the new models. For men, first-degree family history and the 140-SNP PRS were potentially problematic in both models (P = 0.05 for the PRS and P = 0.09 for family history for the new multivariable model and both P < 0.001 for the new family history and PRS model. Plots of the Schoenfeld residuals in S2 Fig showed no strong trend with age for any of the potentially problematic variables and we chose to proceed without age interactions.

Table 3 shows the risk factors for the new multivariable models and the new family history and PRS models expressed as the hazard ratio per adjusted SD [42] to allow direct comparison of the strength of the associations.

**Table 3:** Hazard ratios per adjusted standard deviation for the risk factors in the new models for women and men in the 70% training dataset.

Risk factor	Hazard ratio per adjusted SD	95% confidence interval	P value
**Women – new multivariable model**			
140-SNP PRS (standardised)	1.503	1.424, 1.585	<0.001
Affected first-degree relative, any	1.086	1.035, 1.140	0.001
Smoking, ever	1.114	1.056, 1.174	<0.001
Screening procedure in last 10 years, yes	0.879	0.824, 0.938	<0.001
Triglycerides (mmol/L, centred)	1.082	1.028, 1.140	0.001
**Women – new family history and PRS model**			
140-SNP PRS (standardised)	1.497	1.420, 1.580	<0.001
Affected first-degree relative, any	1.028	0.975, 1.083	0.3
**Men – new multivariable model**			
140-SNP PRS (standardised)	1.484	1.417, 1.553	<0.001
Affected first-degree relative, any	1.126	1.082,1.173	<0.001
Smoking, ever	1.177	1.122, 1.235	<0.001
Screening procedure in last 10 years, yes	0.911	0.864, 0.961	<0.001
Body mass index (natural log of kg/m^2^, centred)	1.153	1.101, 1.207	<0.001
**Men – new family history and PRS model**			
140-SNP PRS (standardised)	1.479	1.413, 1.549	<0.001
Affected first-degree relative, any	1.120	1.074, 1.168	<0.001

Note: PRS, polygenic risk score; SD standard deviation.

### Testing

Summary statistics for the 10-year risk scores for women and men in the 30% testing dataset are shown in Table 4. The interquartile ranges were similar for the models for women and for the models for men but were 80–90% greater for men than for women. Table 5 shows the performance of the models in terms of association with colorectal cancer, discrimination and calibration. The 10-year risks for all models were strongly associated with colorectal cancer. For women, the current family history and PRS model, the new family history and PRS model, and the new multivariable model all had a higher HR per SD than the average risk model (P = 0.04, 0.001 and < 0.001, respectively) and the family history alone model (all P < 0.001). For men, the current family history and PRS model, the new family history and PRS model, and the new multivariable model all had a higher HR per SD of 10-year risk than both the average risk model and the family history alone model (all P < 0.001). For women and men, there was no difference in the HR per SD of 10-year risk for the new family history and PRS model and the new multivariable model (P = 0.3 for women and P = 0.2 for men); the new family history and PRS model and the new multivariable model both has a stronger association than the current family history and PRS model (both P < 0.001 for women and men). There was no difference in the strength of association between women and men for any of the models (all P > 0.3).

**Table 4:** Summary statistics for 10-year risk scores (%) in the 30% testing dataset.

	Mean	SD	Median	IQR	Min	Max
**Women**						
Average risks	0.93	0.50	0.94	0.91	0.13	1.88
Family history alone model	0.98	0.67	0.91	0.85	0.12	3.89
Current family history and PRS model	0.96	0.87	0.74	0.88	0.02	12.68
New family history and PRS model	1.01	0.73	0.86	0.95	0.02	8.00
New multivariable model	1.04	0.79	0.85	0.99	0.02	10.36
**Men**						
Average risks	1.54	0.89	1.62	1.75	0.16	2.93
Family history alone model	1.63	1.17	1.62	1.64	0.14	6.05
Current family history and PRS model	1.58	1.46	1.23	1.58	0.03	27.52
New family history and PRS model	1.67	1.25	1.45	1.72	0.05	14.72
New multivariable model	1.72	1.39	1.43	1.82	0.04	14.47

Note: IQR, interquartile range; Max, maximum; Min, minimum; PRS, polygenic risk score; SD, standard deviation.

**Table 5:** Performance of 10-year risk prediction scores in the 30% testing dataset.

Association	Hazard ratio per SD	95% confidence interval	P value
**Women**			
Average risks	1.453	1.079, 1.956	0.01
Family history alone model	1.357	1.138, 1.617	0.001
Current family history and PRS model	1.864	1.650, 2.107	<0.001
New family history and PRS model	2.172	1.871, 2.522	<0.001
New multivariable model	2.233	1.935, 2.577	<0.001
**Men**			
Average risks	1.112	0.845, 1.465	0.4
Family history alone model	1.220	1.023, 1.456	0.03
Current family history and PRS model	1.949	1.732, 2.193	<0.001
New family history and PRS model	2.342	2.024, 2.710	<0.001
New multivariable model	2.432	2.119, 2.791	<0.001
**Discrimination**	**Harrell’s** **C-index**	**95% confidence interval**	**P value** ^*^
**Women**			
Average risks	0.643	0.621, 0.664	<0.001
Family history alone model	0.643	0.621, 0.664	<0.001
Current family history and PRS model	0.683	0.663, 0.703	<0.001
New family history and PRS model	0.683	0.663, 0.704	<0.001
New multivariable model	0.690	0.669, 0.712	<0.001
**Men**			
Average risks	0.642	0.624, 0.660	<0.001
Family history alone model	0.641	0.623, 0.659	<0.001
Current family history and PRS model	0.689	0.671, 0.707	<0.001
New family history and PRS model	0.692	0.673, 0.710	<0.001
New multivariable model	0.699	0.681, 0.717	<0.001
**Calibration – slope**	**β**	**95% confidence interval**	**P value** ^**^
**Women**			
Average risks	0.879	0.718, 1.041	0.1
Family history alone model	0.735	0.606, 0.865	0.001
Current family history and PRS model	0.792	0.686, 0.898	<0.001
New family history and PRS model	0.947	0.818, 1.075	0.4
New multivariable model	0.939	0.816, 1.062	0.3
**Men**			
Average risks	0.777	0.652, 0.902	<0.001
Family history alone model	0.666	0.563, 0.770	<0.001
Current family history and PRS model	0.766	0.677, 0.854	<0.001
New family history and PRS model	0.903	0.796, 1.010	0.08
New multivariable model	0.892	0.791, 0.993	0.04
**Calibration – intercept**	**α**	**95% confidence interval**	**P value**
**Women**			
Average risks	−0.100	−0.184, −0.015	0.02
Family history alone model	−0.154	−0.239, −0.070	<0.001
Current family history and PRS model	−0.132	−0.217, −0.047	0.002
New family history and PRS model	−0.185	−0.270, −0.101	<0.001
New multivariable model	−0.209	−0.294, −0.124	<0.001
**Men**			
Average risks	−0.151	−0.225, −0.078	<0.001
Family history alone model	−0.210	−0.284, −0.136	<0.001
Current family history and PRS model	−0.182	−0.256, −0.108	<0.001
New family history and PRS model	−0.234	−0.308, −0.161	<0.001
New multivariable model	−0.269	−0.344, −0.196	<0.001

Note: * for test that Harrell’s C-index = 0.5; ** for test that β = 1.

For discrimination, the new models performed well for both women and men, as did the current family history and PRS model (Table 5). For women, the estimate of Harrell’s C-index was higher for the new multivariable model compared with the new family history and PRS model (P = 0.02) but there was no difference between the discrimination of the current family history and PRS model and both the new multivariable model (P = 0.1) and the new family history and PRS model (P = 0.9). For men, the new multivariable model discriminated better than the current family history and PRS model (P = 0.008) and the new family history and PRS model (P = 0.01). There was no difference in discrimination between the current family history and PRS model and the new family history and PRS model (P = 0.3). These three models all discriminated better than the average risk model and the family history alone model for women and men (all P < 0.001). There were no differences in discrimination between women and men (all P > 0.6).

For women, the calibration slopes were close to 1 for the average risk model, the new family history and PRS model, and the new multivariable model but not for the family history alone model and the current family history and PRS model. For men, the slope for the new family history and PRS model was close to 1, while the slope for the new multivariable model was slightly diminished (Table 5). For women and men, both new models were a marked improvement over the family history alone model and the current family history and PRS model (all P < 0.001). For women, the slope of the average risks was not different from the slopes of the new multivariable model (P = 0.3) or the new family history and PRS model (P = 0.2), while for men the slope of the average risks was worse than the slopes for the new multivariable model (P = 0.003) and the new family history and PRS model (P = 0.006).

**Fig 1:**
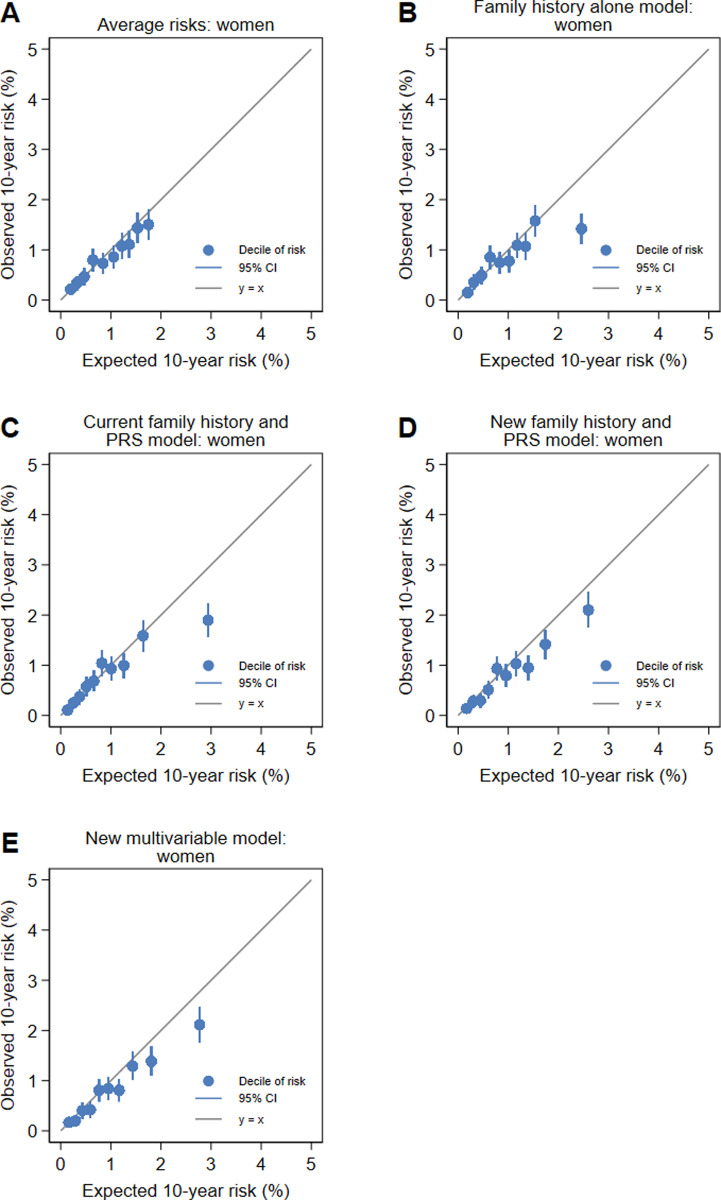
Calibration plots for the (A) average risks, (B) family history alone model, (C) current family history and PRS model, (D) new family history and PRS model and (E) new multivariable model for women.

**Fig 2:**
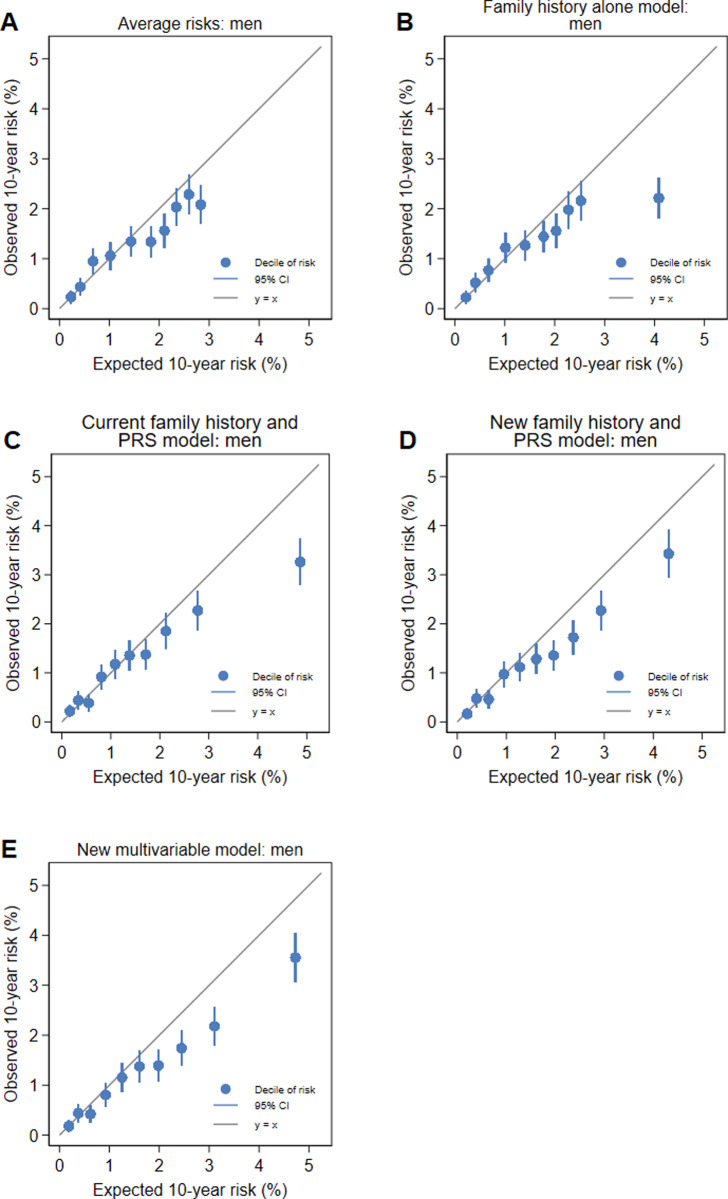
Calibration plots for the (A) average risk, (B) family history alone model, (C) current family history and PRS model, (D) new family history and PRS model and (E) new multivariable model for men.

There were no differences in calibration slope between women and men for each of the models (all P > 0.2), except for the family history alone model, for which there was marginal evidence that the slopes differed (P = 0.06), with the slope for women being close to 1 than the slope for men. The calibration plots for women in Fig 1 and for men in Fig 2 show that miscalibration is generally only evident in the top two or three deciles of risk. This would result in the overall overestimation of risk seen in the calibration slopes and the intercepts. The estimation of risk is well calibrated for those at low or average risk and, in some cases, overestimates risk for those at increased risk. The intercepts for all models for men and women were all below 0 (Table 5), with the intercepts for the new multivariable model being lower than the intercept for the other models for both women and men (all P < 0.001). There were no differences in the intercepts between women and men for all models (all P > 0.3).

**Fig 3:**
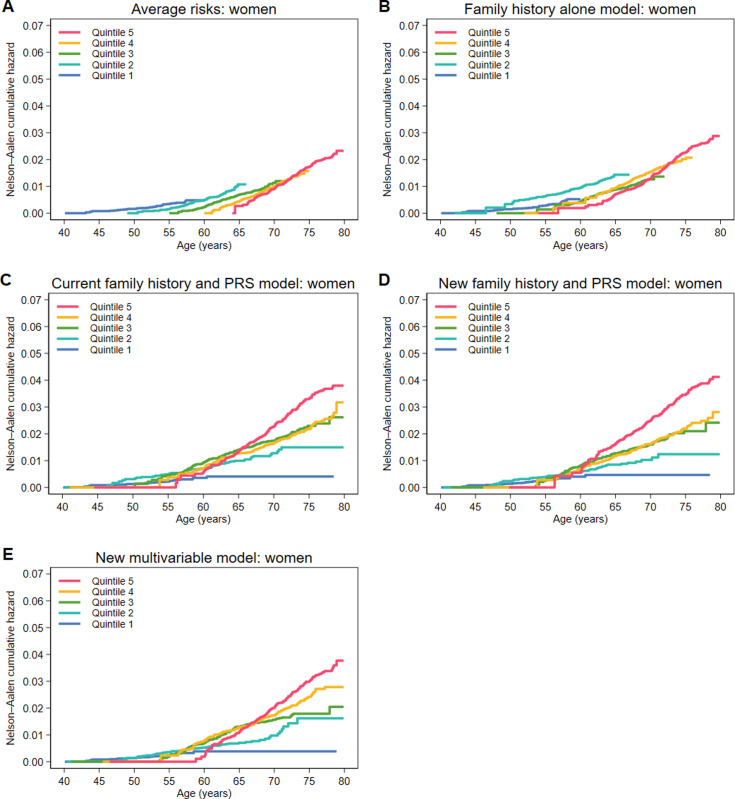
Nelson–Aalen cumulative hazard curves stratified by quintile of 10-year risk from age 40 to 80 years for women for the (A) average risks, (B) family history alone model, (C) current family history and PRS model, (D) new family history and PRS model and (E) new multivariable model.

**Fig 4:**
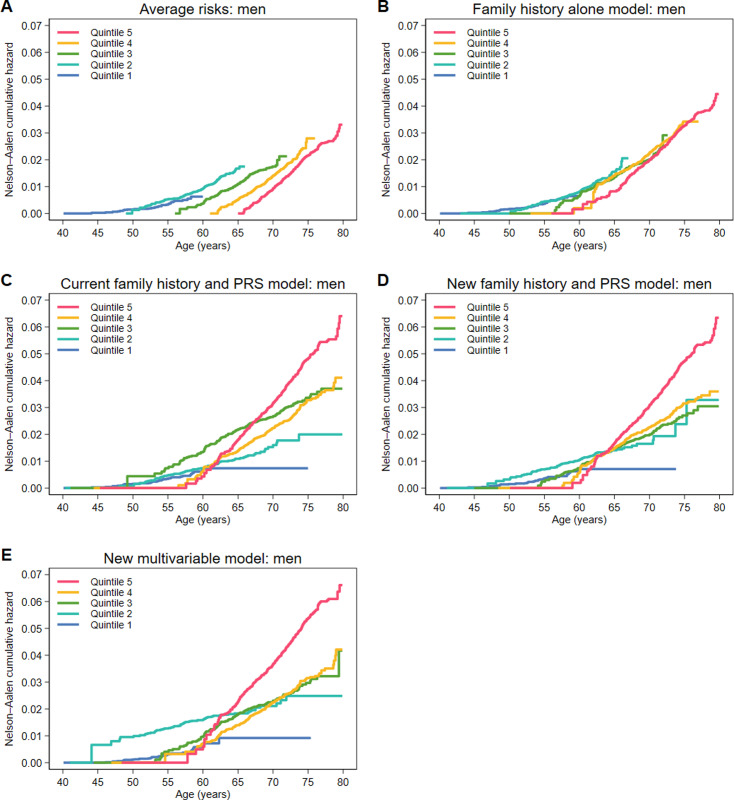
Nelson–Aalen cumulative hazard curves stratified by quintile of 10-year risk from age 40 to 80 years for men for the (A) average risks, (B) family history alone model, (C) current family history and PRS model, (D) new family history and PRS model and (E) new multivariable model.

For each of the risk models, and for women and men separately, graphs of the Nelson–Aalen cumulative hazards from age 40–80 years are shown in Fig 3 for women and Fig 4 for men, and the corresponding cumulative hazards at aged 55, 65 and 75 years are presented in S4 Table. For the average risks (and to a lesser extent for the family history alone model), an individual’s age determines their quintile of risk and trajectory of cumulative risk. For the other models, the top quintile of risk identifies individuals who have a high cumulative risk in later life. For women, the cumulative risks for quintiles 3 and 4 for the new family history and PRS model overlap substantially after age 65 years, while for the new multivariable model, the cumulative risks for quintile 4 are between those for quintile 3 and quintile 5 after age 75 years. For men, the middle three quintiles overlap for both of the new models and the top quintile identifies individuals at substantially increased cumulative risk. For quintile 2, the cumulative risks to age 75 years are higher for men than for women. In the new models and in the current family history and PRS model, quintile 1 identifies individuals at very low risk for both women and men.

Table 6 shows the performance of the models stratified by 10-year age group with comparisons between age groups. Notably, the HRs per SD were consistent across age groups for the new multivariable model for women but the current family history and PRS model and the new family history and PRS models had a higher HR per SD in the 40–49 years age group than in the 60–69 years age group (the current family history and PRS model also had a higher HR per SD in the 40–49 years age group than in the 50–59 year age group). No differences in association by age group were seen for men.

**Table 6:** Performance of 10-year risk prediction scores in the 30% testing dataset by 10-year age group with comparisons with other age groups.

Association	Hazard ratio per SD	95% confidence interval	P value	χ^2^ (df = 1)	P value
**Women**					
***Age 40–49 years***				Compared with 50–59 years	
Average risks	1.422	0.504, 4.007	0.5	0.00	0.9
Family history alone model	2.608	1.447, 4.699	0.001	3.34	0.07
Current family history and PRS model	3.629	2.358, 5.586	<0.001	7.08	0.008
New family history and PRS model	3.875	2.249, 6.678	<0.001	2.20	0.1
New multivariable model	2.664	1.575, 4.509	<0.001	0.25	0.6
***Age 50–59 years***				Compared with 60–69 years	
Average risks	1.324	0.772, 2.272	0.3	1.24	0.3
Family history alone model	1.100	0.778, 1.554	0.6	1.37	0.2
Current family history and PRS model	1.808	1.442, 2.266	<0.001	0.01	0.9
New family history and PRS model	2.212	1.681, 2.911	<0.001	0.05	0.8
New multivariable model	2.203	1.692, 2.869	<0.001	0.12	0.7
***Age 60–69 years***				Compared with 40–49 years	
Average risks	2.280	1.189, 4.372	0.01	0.51	0.5
Family history alone model	1.400	1.100, 1.782	0.006	3.90	0.05
Current family history and PRS model	1.787	1.523, 2.097	<0.001	10.62	0.001
New family history and PRS model	2.137	1.749, 2.611	<0.001	3.79	0.05
New multivariable model	2.349	1.940, 2.844	<0.001	0.15	0.7
**Men**					
***Age 40–49 years***				Compared with 50–59 years	
Average risks	1.271	0.477, 3.387	0.6	0.09	0.8
Family history alone model	1.139	0.552, 2.348	0.7	0.00	1.0
Current family history and PRS model	1.985	1.238, 3.182	0.004	0.05	0.8
New family history and PRS model	2.468	1.404, 4.340	0.002	0.02	0.9
New multivariable model	2.566	1.499, 4.392	0.001	0.01	0.9
***Age 50–59 years***				Compared with 60–69 years	
Average risks	0.959	0.605, 1.522	0.9	0.74	0.4
Family history alone model	1.113	0.802, 1.545	0.5	0.95	0.6
Current family history and PRS model	2.080	1.676, 2.581	<0.001	0.11	0.7
New family history and PRS model	2.525	1.938, 3.289	<0.001	0.00	1.0
New multivariable model	2.598	2.025, 3.333	<0.001	0.00	1.0
***Age 60–69 years***				Compared with 40–49 years	
Average risks	2.524	1.938, 3.289	<0.001	0.23	0.6
Family history alone model	2.598	2.204, 3.333	<0.001	0.05	0.7
Current family history and PRS model	1.851	0.893, 3.838	0.1	0.00	0.9
New family history and PRS model	2.539	2.086, 3.091	<0.001	0.03	0.9
New multivariable model	2.612	2.174, 3.138	<0.001	0.02	0.9
**Discrimination**	**Harrell’s** **C-index**	**95% confidence interval**	**P value** ^**a**^	**z**	**P value**
**Women**					
***Age 40–49 years***				Compared with 50–59 years	
Average risks	0.585	0.502, 0.667	0.04	0.58	0.6
Family history alone model	0.659	0.579, 0.74	<0.001	2.47	0.01
Current family history and PRS model	0.754	0.680, 0.829	<0.001	2.81	<0.001
New family history and PRS model	0.721	0.640, 0.802	<0.001	1.65	0.1
New multivariable model	0.677	0.588, 0.767	<0.001	0.48	0.6
***Age 50–59 years***				Compared with 60–69 years	
Average risks	0.557	0.513, 0.601	0.01	0.01	1.00
Family history alone model	0.544	0.500, 0.587	0.05	0.87	0.4
Current family history and PRS model	0.632	0.591, 0.673	<0.001	0.73	0.5
New family history and PRS model	0.644	0.602, 0.686	<0.001	1.08	0.3
New multivariable model	0.653	0.612, 0.695	<0.001	0.72	0.5
***Age 60–69 years***				Compared with 40–49 years	
Average risks	0.557	0.526, 0.587	0.002	0.62	0.5
Family history alone model	0.567	0.538, 0.597	<0.001	2.10	0.04
Current family history and PRS model	0.613	0.583, 0.643	<0.001	3.45	<0.001
New family history and PRS model	0.615	0.584, 0.646	<0.001	2.39	0.02
New multivariable model	0.634	0.604, 0.664	<0.001	0.90	0.4
**Men**					
***Age 40–49 years***				Compared with 50–59 years	
Average risks	0.620	0.542, 0.699	0.003	1.72	0.09
Family history alone model	0.612	0.538, 0.686	0.003	1.46	0.1
Current family history and PRS model	0.662	0.586, 0.737	<0.001	0.28	0.8
New family history and PRS model	0.682	0.606, 0.758	<0.001	0.71	0.5
New multivariable model	0.688	0.612, 0.764	<0.001	0.54	0.6
***Age 50–59 years***				Compared with 60–69 years	
Average risks	0.543	0.504, 0.582	0.03	0.35	0.7
Family history alone model	0.550	0.511, 0.588	0.01	0.28	0.8
Current family history and PRS model	0.650	0.613, 0.686	<0.001	1.08	0.3
New family history and PRS model	0.651	0.614, 0.689	<0.001	0.82	0.4
New multivariable model	0.665	0.629, 0.701	<0.001	1.05	0.3
***Age 60–69 years***				Compared with 40–49 years	
Average risks	0.551	0.526, 0.576	<0.001	1.64	0.1
Family history alone model	0.556	0.531, 0.582	<0.001	1.40	0.2
Current family history and PRS model	0.625	0.6, 0.65	<0.001	0.90	0.4
New family history and PRS model	0.632	0.607, 0.657	<0.001	1.21	0.2
New multivariable model	0.641	0.616, 0.666	<0.001	1.15	0.3
**Calibration – slope**	**β**	**95% confidence interval**	**P value** ^**b**^	**χ** ^**2**^ ** (df = 1)**	**P value**
**Women**					
***Age 40–49 years***				Compared with 50–59 years	
Average risks	1.030	−0.020, 2.080	1.0	0.22	0.6
Family history alone model	1.321	0.638, 2.004	0.4	6.73	0.01
Current family history and PRS model	1.467	0.999, 1.936	0.05	8.44	0.004
New family history and PRS model	1.652	1.032, 2.273	0.04	2.81	0.09
New multivariable model	1.218	0.641, 1.794	0.5	0.47	0.5
***Age 50–59 years***				Compared with 60–69 years	
Average risks	0.745	0.166, 1.323	0.4	1.33	0.2
Family history alone model	0.354	−0.032, 0.741	0.001	0.57	0.4
Current family history and PRS model	0.712	0.467, 0.957	0.02	0.04	0.8
New family history and PRS model	1.007	0.690, 1.325	1.0	0.04	0.8
New multivariable model	0.965	0.670, 1.261	0.8	0.13	0.7
***Age 60–69 years***				Compared with 40–49 years	
Average risks	1.269	0.577, 1.960	0.4	0.14	0.7
Family history alone model	0.532	0.231, 0.833	0.002	5.02	0.03
Current family history and PRS model	0.682	0.503, 0.862	<0.001	10.09	0.002
New family history and PRS model	0.963	0.722, 1.205	0.8	3.48	0.06
New multivariable model	1.035	0.812, 1.258	0.8	0.26	0.6
**Men**					
***Age 40–49 years***				Compared with 50–59 years	
Average risks	1.151	0.284, 2.017	0.7	1.99	0.2
Family history alone model	0.716	0.094, 1.338	0.4	1.26	0.3
Current family history and PRS model	0.873	0.439, 1.307	0.6	0.15	0.7
New family history and PRS model	1.188	0.642, 1.733	0.5	0.30	0.6
New multivariable model	1.154	0.650, 1.658	0.5	0.32	0.6
***Age 50–59 years***				Compared with 60–69 years	
Average risks	0.458	0.046, 0.871	0.01	5.13	0.02
Family history alone model	0.370	0.064, 0.677	<0.001	0.36	0.54
Current family history and PRS model	0.780	0.573, 0.987	0.04	0.11	0.7
New family history and PRS model	1.017	0.755, 1.280	0.9	0.07	0.8
New multivariable model	0.997	0.758, 1.237	1.0	0.08	0.8
***Age 60–69 years***				Compared with 40–49 years	
Average risks	1.330	0.673, 1.987	0.3	0.11	0.7
Family history alone model	0.489	0.228, 0.749	<0.001	0.58	0.4
Current family history and PRS model	0.738	0.584, 0.892	<0.001	0.35	0.6
New family history and PRS model	1.065	0.855, 1.274	0.5	0.17	0.7
New multivariable model	1.041	0.850, 1.229	0.7	0.18	0.7
**Calibration – intercept**	**α**	**95% confidence interval**	**P value**	**χ** ^**2**^ ** (df = 1)**	**P value**
**Women**					
***Age 40–49 years***				Compared with 50–59 years	
Average risks	0.046	−0.257, 0.349	0.8	0.18	0.7
Family history alone model	0.032	−0.270, 0.336	0.8	0.39	0.5
Current family history and PRS model	0.057	−0.246, 0.360	0.7	0.43	0.5
New family history and PRS model	−0.030	−0.333, −0.273	0.8	0.23	0.6
New multivariable model	−0.027	−0.330, −0.276	0.8	0.39	0.5
***Age 50–59 years***				Compared with 60–69 years	
Average risks	−0.028	−0.183, 0.127	0.7	1.57	0.2
Family history alone model	−0.075	−0.230, 0.080	0.3	1.97	0.2
Current family history and PRS model	−0.057	−0.212, 0.098	0.5	1.79	0.2
New family history and PRS model	−0.114	−0.269, 0.041	0.1	1.57	0.2
New multivariable model	−0.135	−0.291, 0.020	0.09	1.74	0.2
***Age 60–69 years***				Compared with 40–49 years	
Average risks	−0.149	−0.256, −0.042	0.006	1.41	0.2
Family history alone model	−0.210	−0.317, −0.103	<0.001	2.20	0.1
Current family history and PRS model	−0.186	−0.294, −0.079	0.001	2.20	0.1
New family history and PRS model	−0.235	−0.342, −0.128	<0.001	1.55	0.2
New multivariable model	−0.262	−0.369, −0.155	<0.001	2.06	0.2
**Men**					
***Age 40–49 years***				Compared with 50–59 years	
Average risks	0.108	−0.182, 0.398	0.5	0.36	0.5
Family history alone model	0.086	−0.203, 0.376	0.6	0.62	0.4
Current family history and PRS model	0.106	−0.184, 0.396	0.5	0.65	0.4
New family history and PRS model	0.033	−0.257, 0.323	0.8	0.48	0.5
New multivariable model	0.039	−0.250, 0.329	0.8	0.76	0.4
***Age 50–59 years***				Compared with 60–69 years	
Average risks	0.010	−0.128, 0.147	0.9	8.49	0.004
Family history alone model	−0.042	−0.180, 0.095	0.5	9.30	0.002
Current family history and PRS model	−0.026	−0.164, 0.112	0.7	8.12	0.004
New family history and PRS model	−0.080	−0.218, 0.058	0.3	7.94	0.005
New multivariable model	−0.103	−0.241, 0.035	0.1	9.33	0.002
***Age 60–69 years***				Compared with 40–49 years	
Average risks	−0.236	−0.327, −0.145	<0.001	4.92	0.03
Family history alone model	−0.300	−0.391, −0.208	<0.001	6.20	0.01
Current family history and PRS model	−0.267	−0.359, −0.175	<0.001	5.79	0.02
New family history and PRS model	−0.317	−0.409, −0.226	<0.001	5.12	0.02
New multivariable model	−0.360	−0.452, −0.269	<0.001	6.66	0.01

Discrimination followed a similar pattern, being consistent across age groups for the new multivariable model but was higher for the 40–49 years age group than in the 60–69 years age group for the current family history and PRS model and the new family history and PRS models (the current family history and PRS model also had a higher HR per SD in the 40–49 years age group than in the 50–59 year age group). No differences in association by age group were seen for men.

Within the 40–49 years age group for women, the new family history and PRS model discriminated better than the new multivariate model (χ^2 ^= 9.86, degrees of freedom [df] = 1, P = 0.002), with a similar magnitude of difference for the current family history and PRS model (χ^2 ^= 2.07, df = 1, P = 0.2). There was no difference in discrimination between the new multivariable model and the new family history and PRS model for the 50–59 years age group (χ^2 ^= 0.01, df = 1, P = 0.9) but there was for the 60–69 years age group (χ^2 ^= 5.72, df = 1, P = 0.02).

For men in the 40–49 years age group, discrimination was similar for the new multivariable model and the new family history and PRS model (χ^2 ^= 0.13, df = 1, P = 0.7), and the new family history and PRS model discriminated better than the current family history and PRS model (χ^2 ^= 4.49, df = 1, P = 0.03). Similar results were seen for the 50–59 years age group (χ^2 ^= 0.31, df = 1, P = 0.6 and χ^2 ^= 34.23, df = 1, P < 0.001, respectively) and the 60–69 years age group (χ^2 ^= 0.38, df = 1, P = 0.5 and χ^2 ^= 39.17, df = 1, P < 0.001, respectively).

Table 7 shows for women and men, the SIRs compared to population incidence rates overall, by 10-year age group, for the first four quintiles and the top two deciles of risk for each of the models, and for first-degree family history. Overall, for both women and men, fewer colorectal cancers were observed than the number expected using sex- and age-specific population incidence rates. Breaking this down by 10-year age group showed that this finding was driven by the 60–69 years age group.

**Table 7:** Standardised incidence ratios overall and by 10-year age group, for the first four quintiles and the top two deciles of risk for each of the models, and by first-degree family history.

	Observed	Expected	SIR	95% confidence interval
**Women**				
Overall	543	597.1	0.910	0.836, 0.989
40–49 years	42	43.9	0.956	0.706, 1.293
50–59 years	161	164.4	0.839	0.839, 1.143
60–69 years	340	388.7	0.787	0.787, 0.973
**Average risks**				
Quintile 1 (median = 0.3%)	37	38.6	0.960	0.695, 1.325
Quintile 2 (median = 0.6%)	79	73.6	1.074	0.862, 1.339
Quintile 3 (median = 0.9%)	106	120.5	0.880	0.727, 1.064
Quintile 4 (median = 1.3%)	140	155.9	0.898	0.761, 1.060
Decile 9 (median = 1.5%)	89	95.6	0.931	0.756, 1.146
Decile 10 (median = 1.7%)	92	113.0	0.815	0.664, 0.999
**Family history alone model**				
Quintile 1 (median = 0.2%)	33	39.1	0.844	0.600, 1.187
Quintile 2 (median = 0.6%)	85	77.2	1.102	0.891, 1.363
Quintile 3 (median = 0.9%)	96	115.9	0.828	0.678, 1.011
Quintile 4 (median = 1.3%)	145	171.5	0.845	0.719, 0.995
Decile 9 (median = 1.5%)	98	104.3	0.940	0.771, 0.146
Decile 10 (median = 2.4%)	86	89.1	0.965	0.781, 1.193
**Current family history and PRS model**				
Quintile 1 (median = 0.2%)	23	44.5	0.517	0.343, 0.777
Quintile 2 (median = 0.4%)	61	87.8	0.695	0.540, 0.893
Quintile 3 (median = 0.7%)	111	129.2	0.859	0.713, 1.035
Quintile 4 (median = 1.1%)	124	157.2	0.789	0.662, 0.941
Decile 9 (median = 1.6%)	102	86.2	1.184	0.975, 1.438
Decile 10 (median = 2.6%)	122	92.1	1.324	1.109, 1.582
**New family history and PRS model**				
Quintile 1 (median = 0.2%)	27	42.5	0.635	0.435, 0.926
Quintile 2 (median = 0.5%)	52	83.5	0.623	0.475, 0.817
Quintile 3 (median = 0.8%)	111	126.8	0.875	0.727, 1.054
Quintile 4 (median = 1.3%)	127	158.2	0.803	0.675, 0.955
Decile 9 (median = 1.7%)	91	88.6	1.027	0.837, 1.262
Decile 10 (median = 2.4%)	135	97.5	1.385	1.170, 1.640
**New multivariable model**				
Quintile 1 (median = 0.2%)	24	43.8	0.549	0.368, 0.818
Quintile 2 (median = 0.5%)	53	85.7	0.618	0.472, 0.809
Quintile 3 (median = 0.9%)	106	126.6	0.837	0.692, 1.013
Quintile 4 (median = 1.3%)	135	156.94	0.860	0.727, 1.018
Decile 9 (median = 1.8%)	89	88.1	1.010	0.821, 1.244
Decile 10 (median = 2.5%)	136	95.9	1.418	1.199, 1.678
**Affected first-degree relative**				
No	463	528.4	0.876	0.800, 0.960
Yes	80	68.7	1.165	0.936, 1.451
**Men**				
Overall	723	821.5	0.880	0.818, 0.947
40–49 years	46	46.1	0.999	0.748, 1.334
50–59 years	206	204.3	1.008	0.880, 1.156
60–69 years	471	571.1	0.825	0.754, 0.903
**Average risks**				
Quintile 1 (median = 0.3%)	38	41.0	0.928	0.675, 1.275
Quintile 2 (median = 0.8%)	107	95.3	1.123	0.929, 1.358
Quintile 3 (median = 1.6%)	151	172.6	0.875	0.746, 1.026
Quintile 4 (median = 2.2%)	191	217.9	0.876	0.761, 1.010
Decile 9 (median = 2.6%)	124	137.7	0.901	0.755, 1.074
Decile 10 (median = 2.8%)	112	157.1	0.713	0.593, 0.858
**Family history alone model**				
Quintile 1 (median = 0.3%)	41	41.8	0.982	0.723, 1.334
Quintile 2 (median = 0.8%)	106	98.3	1.078	0.891, 1.304
Quintile 3 (median = 1.6%)	155	179.2	0.865	0.739, 1.013
Quintile 4 (median = 2.2%)	186	226.0	0.823	0.713, 0.950
Decile 9 (median = 2.5%)	117	149.8	0.781	0.652, 0.936
Decile 10 (median = 4.2%)	118	126.4	0.934	0.780, 1.119
**Current family history and PRS model**				
Quintile 1 (median = 0.2%)	36	46.3	0.777	0.561, 1.078
Quintile 2 (median = 0.7%)	71	115.0	0.618	0.489, 0.779
Quintile 3 (median = 1.2%)	138	186.7	0.739	0.626, 0.873
Quintile 4 (median = 1.9%)	176	225.5	0.780	0.673, 0.905
Decile 9 (median = 2.7%)	124	121.0	1.025	0.860, 1.222
Decile 10 (median = 4.3%)	178	127.0	1.402	1.210, 1.624
**New family history and PRS model**				
Quintile 1 (median = 0.3%)	35	43.7	0.801	0.575, 1.115
Quintile 2 (median = 0.8%)	78	108.1	0.722	0.578, 0.901
Quintile 3 (median = 1.4%)	131	184.8	0.709	0.597, 0.841
Quintile 4 (median = 2.2%)	168	227.2	0.740	0.636, 0.860
Decile 9 (median = 2.9%)	124	125.4	0.989	0.830, 1.180
Decile 10 (median = 4.0%)	187	132.4	1.413	1.224, 1.631
**New multivariable model**				
Quintile 1 (median = 0.3%)	34	45.4	0.749	0.535, 1.049
Quintile 2 (median = 0.8%)	67	111.8	0.599	0.472, 0.762
Quintile 3 (median = 1.4%)	138	184.5	0.748	0.633, 0.884
Quintile 4 (median = 2.2%)	171	226.4	0.755	0.650, 0.877
Decile 9 (median = 3.1%)	119	122.9	0.968	0.809, 1.159
Decile 10 (median = 4.4%)	194	130.4	1.487	1.292, 1.712
**Affected first-degree relative**				
No	618	723.3	0.854	0.780, 0.925
Yes	105	98.2	1.069	0.883, 1.295

**Fig 5:**
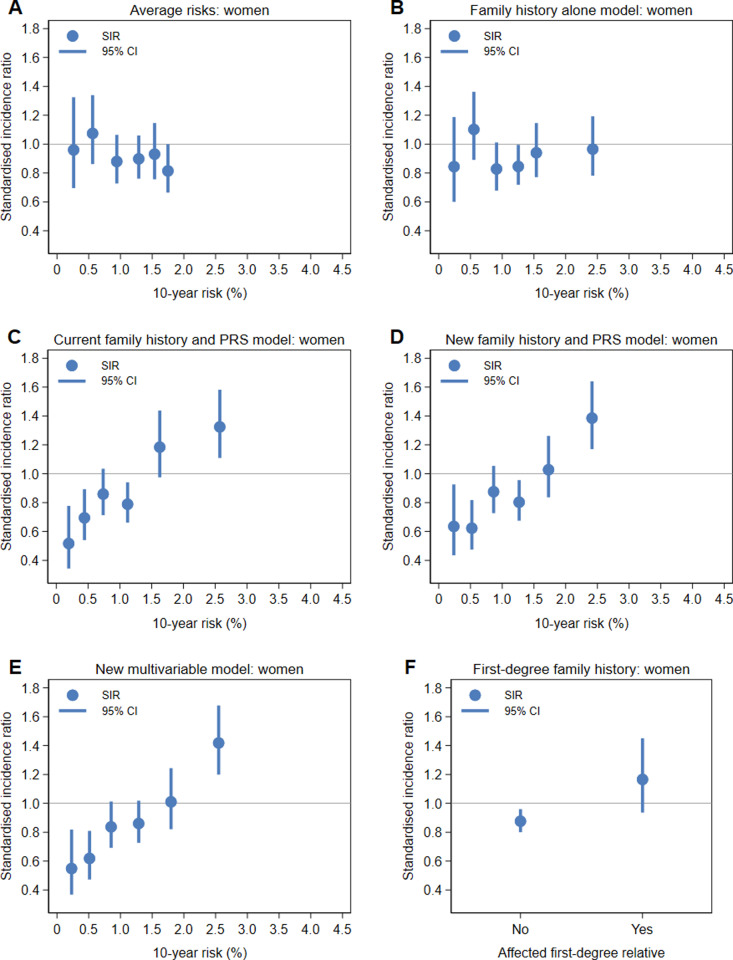
Standardised incidence ratios compared to population incidence rates for the first four quintiles and the top two deciles of risk for women for the (A) average risks, (B) family history alone model, (C) current family history and PRS model, (D) new family history and PRS model and (E) new multivariable model, and for (F) the absence and presence of a first-degree family history of colorectal cancer.

**Fig 6:**
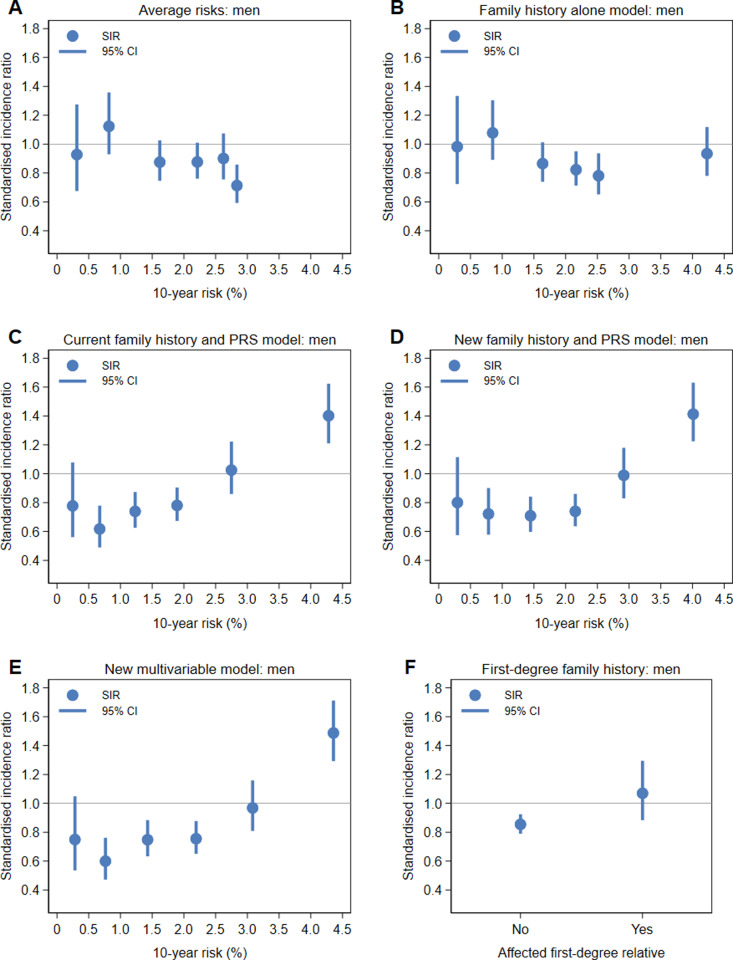
Standardised incidence ratios compared to population incidence rates for the first four quintiles and the top two deciles of risk for men for the (A) average risks, (B) family history alone model, (C) current family history and PRS model, (D) new family history and PRS model and (E) new multivariable model, and for (F) the absence and presence of a first-degree family history of colorectal cancer.

For each of the models, the SIRs from Table 7 are plotted on the *x*-axis at the median value of the quintile or decile for women and men in Figs 5 and 6, respectively. For all models, the risk predictions for men stratified risk more than the risk predictions for women. For example, the median risk for the top decile of the new multivariable model was 2.5% for women and 4.4% for men, with similar differences for the other models. For the average risks and family history alone model in both women and men, none of the risk groups were at increased risk of colorectal cancer. For the current family history and PRS model and for the new models, the top decile of risk identified individuals at increased risk of colorectal cancer, with the SIRs ranging from 1.3–1.4 for women and 1.4–1.5 for men. While for each of the new models, the second top decile was not at increased risk, the SIRs were higher (albeit not statistically significantly so) than those for the four quintiles of risk, as was having an affected first-degree relative for women.

## Discussion

Current colorectal cancer risk stratification is based on hereditary cancer syndrome, family history of colorectal cancer, and a personal history of inflammatory bowel disease. However, in the absence of these risk factors, a significant number of adults – who appear to be at average risk – go on to develop colorectal cancer. We previously developed a risk prediction model for the general population that incorporates first-degree family history of colorectal cancer and a PRS comprising 140 SNPs [25]. Because several clinical risk factors are strongly associated with colorectal cancer risk, we sought to improve upon our current model by developing, separately for women and men, two models: (i) a reparameterisation of the previous family history and 140-SNP PRS model and (ii) a multivariable model. We then compared the performance of these new models to predictions from our current family history and PRS model, a family history alone model and average risks.

In identifying a preferred model, the two new models and the current family history and PRS model outperformed the risk predictions based on average risks and family history alone. The new multivariable model had better discrimination than the new family history and PRS model for both women and men (P = 0.02 and P = 0.01, respectively). This improvement in performance for the multivariable models came from the inclusion of three additional risk factors (ever smoked, colorectal cancer screening in the past 10 years, and triglyceride level [women] or body mass index [men]).

The discriminatory benefit of the new multivariable model was seen in women aged 60–69 years (P = 0.02), whereas, in women aged 40–49 years, the new family history and PRS model showed similar discrimination to the current family history and PRS model (P = 0.7) and discriminated better than the new multivariable model (P = 0.002). There was no difference in discrimination between these models in the 50–59 years age group or for men in any age group. While this may be a chance finding, and should be confirmed in future studies, it presents implications for clinical care where, for women (in whom risk prediction has often performed worse than for men [17–19]) the simpler model may be beneficial to tailor earlier screening recommendations to women aged under 50 years, whereas the multivariable model may be beneficial to tailor on-going screening intervals, screening modality, compliance and even screening cessation in women aged 60 years and over. For men, either model could be used.

The assessment of calibration gives additional insight into identifying a preferred model. For men, the calibration slope for the new family history and PRS model was close to 1 while the slope for the new multivariable model was not. The intercepts for calibration must be interpreted in the context of the well-known healthy volunteer effect of the UK Biobank [31]. We observed fewer colorectal cancers than expected using population incidence rates: for women, there were 9% fewer colorectal cancers than expected and for men there were 12% fewer colorectal cancers than expected (Table 7). If the healthy volunteer effect was not present, these intercepts might have been closer to zero. The calibration plots in Figs 1 and 2 are reassuring because the four lowest quintiles of risk are well calibrated for both models for women and for men. The risks for low-risk and average risk individuals will be accurate.

We developed separate models for women and men because of previous findings that colorectal cancer risk prediction better for men than for women [17–19]. We found that the range of risk stratification was wider for men than for women, with an interquartile range around 80% greater for men than for women for both of the models (Table 4).

The SIRs compared to population incidence rates (Table 7, Figs 5 and 6) clearly show that individuals in the top decile of risk for the new models are at increased risk of colorectal cancer whereas those with a first-degree family history are not. These results might be lower than what would be expected if the UK Biobank’s healthy volunteer effect [31] was not present. Overall, there were about 10% fewer colorectal cancers observed than the number expected using population incidence rates. If the cohort has as many cases as had been expected, the second highest deciles of risk might also have been at increased risk.

The family history alone model performed poorly. This is not surprising because fewer than 10% of the population have a first-degree family history of colorectal cancer [7] and, therefore, the other 80% of the population would only have their age taken into account for risk prediction. Direct comparison of the strength of association for the risk factors (Table 3) showed that the PRS was by far the strongest risk factor and that first-degree family history was the second-weakest risk factor for both women and men. The PRS also has advantages because it is measured on a continuous scale and is measured for everyone, unlike first-degree family history. By including the PRS in the risk prediction models, we can identify individuals who are at substantially increased risk but do not have a first-degree family history.

While we have demonstrated the added value of a few clinical risk factors (smoking, triglycerides [for women], body mass index [for men] and colorectal cancer screening) in a multivariable model with family history and PRS, it is important to consider the feasibility of implementing the models at a population level. These risk factors are typically already within a patient’s electronic medical record and pulling them into an algorithm to calculate risk can be done without impeding on a clinician’s time. If implemented at an institutional level, this would be a simple, yet valuable, risk stratification tool for population healthcare that addresses both the non-compliant screeners and the increased incidence in early onset colorectal cancer.

One important limitation of this study is that the PRS we have used was developed in a dataset that included the UK Biobank (which represented 10% of cases and 33% of controls in the derivation dataset) [26]. This may mean that the performances of the models using the PRS are overestimated to a small degree but will not affect the comparisons between models. Another limitation is that the UK Biobank does not have sufficient participants of ancestries other than United Kingdom to undertake meaningful analyses. An important next step will be to validate the models developed here in large datasets comprising individuals of other ancestries and, where possible, using ancestry-specific incidence rates. We were unable to distinguish between different types of colorectal cancer screening (colonoscopy vs faecal immunochemical test). Being able to do so might have enabled separate risks to be calculated for the different modes of screening.

We studied 10-year risks to make maximum use of the available follow-up time, but the equations can be used to predict risk over other periods, such as, 5-year risk, full-lifetime risk or remaining lifetime risk. Using our risk prediction models, we can identify individuals who are at substantially increased risk, even if they do not have a first-degree family history. Previously, these individuals would have been missed and not able to avail themselves of additional screening appropriate for people at increased risk.

## Supporting information

S1 Fig Nelson–Aalen cumulative hazard function and Cox–Snell residuals from the first imputation dataset for the new family history and PRS model for (A) women and (B) men, and for the new multivariable model for (C) women and (D) men. (PDF)

S2 Fig Scaled Schoenfeld residuals by age in the first imputation dataset for (A) first-degree family history and (B) 140-SNP polygenic risk score in the new family history and PRS model for men and for (C) first-degree family history and (D) 140-SNP polygenic risk score in the new multivariable model for men. (PDF)

S1 Table UK Biobank data fields used to derive variables for analysis and eligibility assessment. (PDF)

S2 Table Summary statistics for unaffected and affected women and men for baseline risk factors considered in the development of the colorectal cancer risk prediction models. (PDF)

S3 Table Unadjusted hazard ratios for women and men for the baseline risk factors considered in the development of the colorectal cancer risk prediction models using the multiple imputation data for the 70% training dataset. (PDF)

S4 Table Nelson–Aalen cumulative hazards extracted at 5-year intervals from age 40 to 80 years separately for women and men for the risk prediction models. (PDF)
