# Supplementary material for: Colorectal cancer risk prediction using a simple multivariable model
Source: PLoS One. 2025 May 13;20(5):e0321641. doi: 10.1371/journal.pone.0321641 (PMC12074527; doi:10.1371/journal.pone.0321641)
Supplement: S1 Fig — (PDF) [file pone.0321641.s001.pdf]

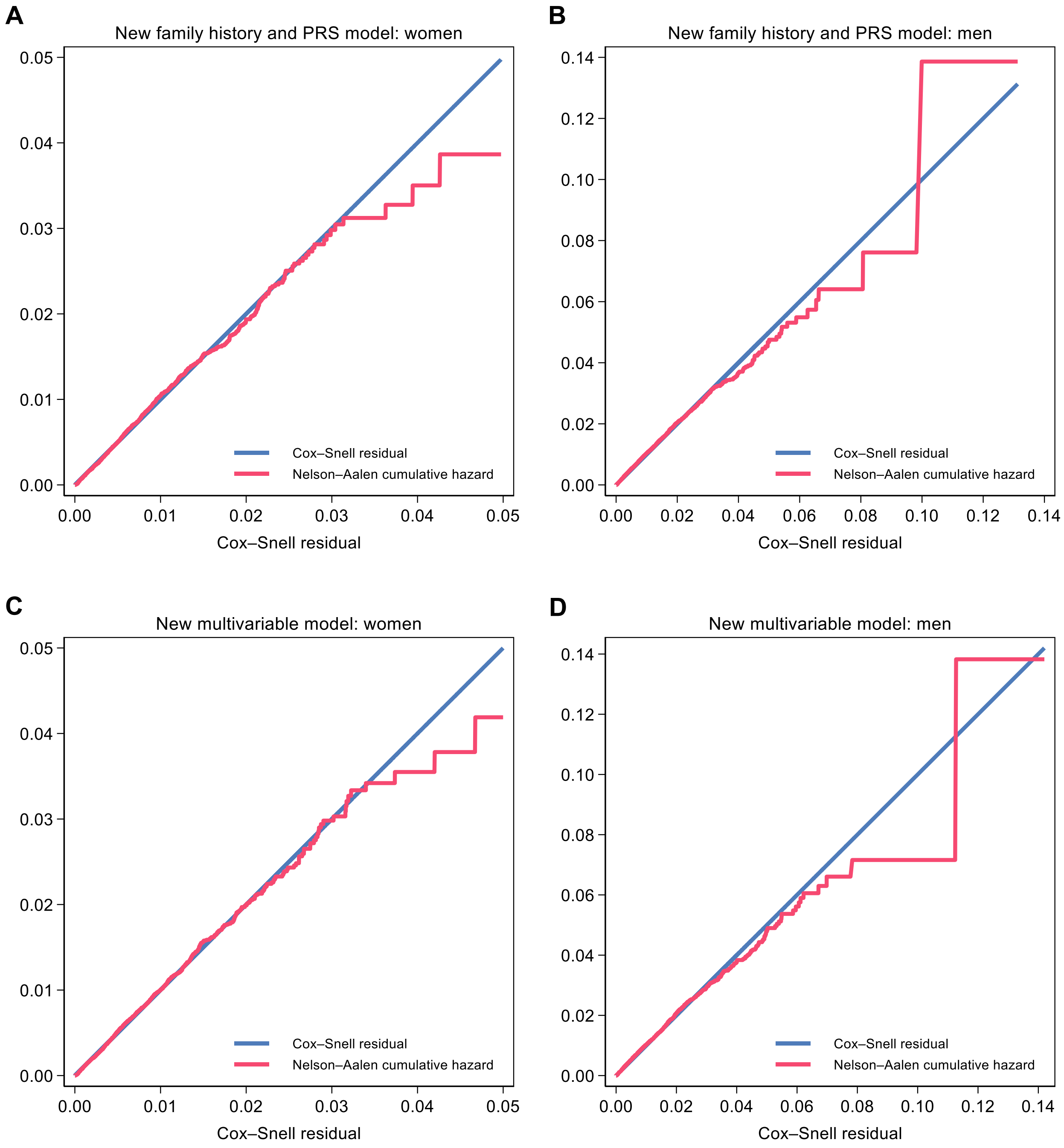

S1 Figure. Nelson–Aalen cumulative hazard function and Cox–Snell residuals from the first imputation dataset for the new family history and PRS model for (A) women and (B) men, and for the new multivariable model for (C) women and (D) men.
