# Supplementary material for: Colorectal cancer risk prediction using a simple multivariable model
Source: PLoS One. 2025 May 13;20(5):e0321641. doi: 10.1371/journal.pone.0321641 (PMC12074527; doi:10.1371/journal.pone.0321641)
Supplement: S2 Fig — (PDF) [file pone.0321641.s002.pdf]

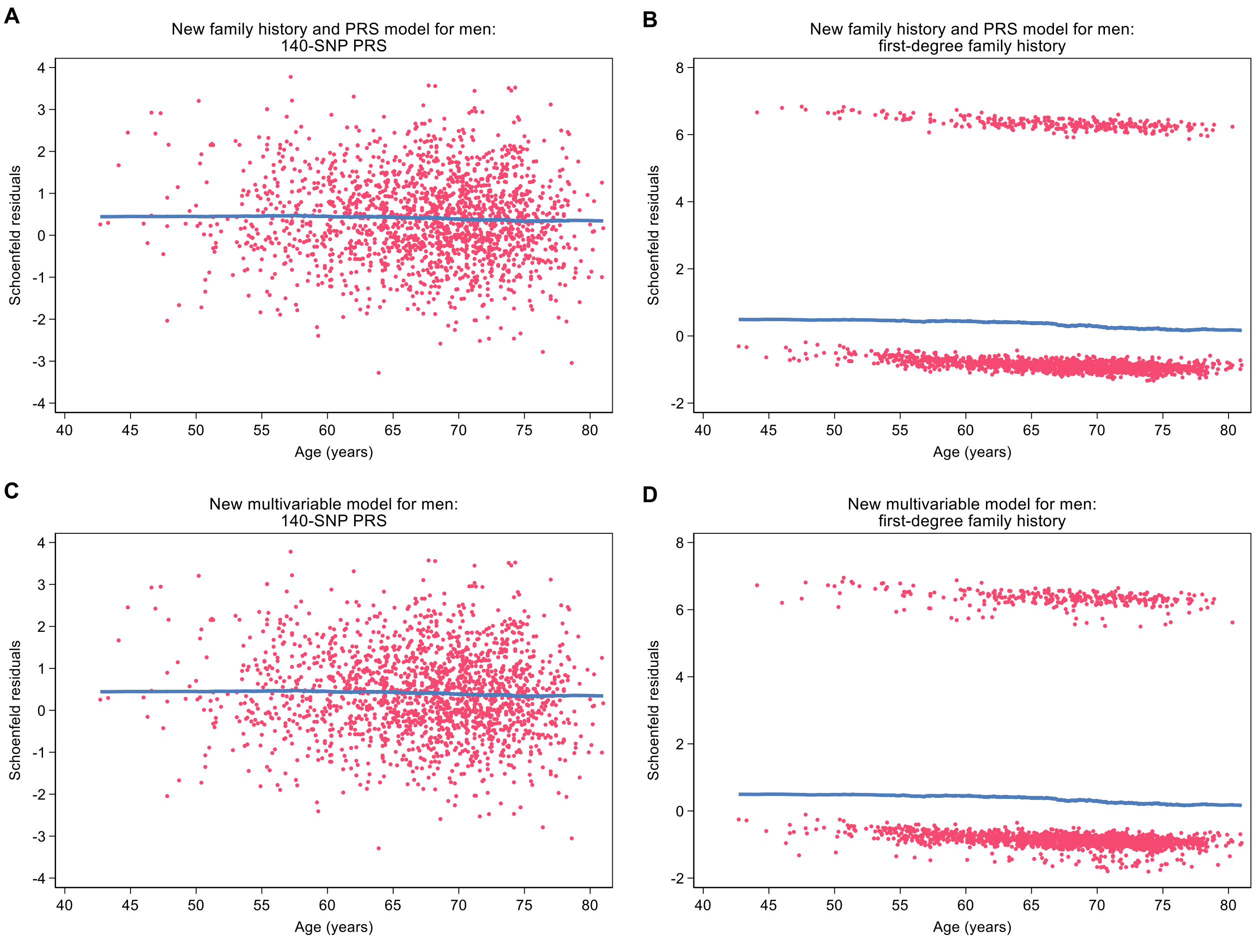

S2 Figure. Scaled Schoenfeld residuals by age in the first imputation dataset for (A) first-degree family history and (B) 140-SNP polygenic risk score in the new family history and PRS model for men and for (C) first-degree family history and (D) 140-SNP polygenic risk score in the new multivariable model for men.
