# Supplementary material for: Colorectal cancer risk prediction using a simple multivariable model
Source: PLoS One. 2025 May 13;20(5):e0321641. doi: 10.1371/journal.pone.0321641 (PMC12074527; doi:10.1371/journal.pone.0321641)
Supplement: S1 Table — (PDF) [file pone.0321641.s003.pdf]

S1 Table. UK Biobank data fields used to derive variables for analysis and eligibility assessment

Variable	Data fields	Related age or date fields	Note
Age at baseline assessment	21003	34, 52, 53	Calculated from baseline assessment date and month and year of birth
Genetic sex/gender identity	22001, 31		
Colorectal cancer diagnosis	20001, 40006, 40013	20006, 20007, 40005, 40008	20001 = 1020, 1022, 1023; 40006 = C18*, C19*, C20*; 40013 = 153*, 1540, 1541
Age at death	40000	40007	
First-degree family history of colorectal cancer	20107, 20110, 20111		Mother, 20110 = 4; father, 20107 = 4, sibling, 20111 = 4; there is no way of knowing if more than one sibling is affected
Body mass index	21001		
Polyps	20002, 20004, 41270, 41272	20008, 20010, 20011 41280, 41282	20002 = 1460; 20004 = 1463; 41270 = K621, K635; 41272 = H20*, H23*, H26*
Chron's disease	131626		Before baseline assessment date
Ulcerative colitis	131628		Before baseline assessment date
Type 2 or unspecified diabetes	130708, 130714		Before baseline assessment date
Screening procedure for colorectal cancer	20004, 41272	20010, 20011, 41280, 41282	20004 = 1463, 1519; 41272 = H20*, H22*, H23*, H25*, H26*, H28* (before baseline assessment date)
High-density lipoprotein	30760		
Triglycerides	30870		
Low-density lipoprotein	30780		
Total cholesterol	30960		

Variable	Data fields	Related age or date fields	Note
Non-steroidal anti-inflammatory drug use	6154, 20003		6154 = 1, 2; 20003 = 140861806, 1140861808, 1140864860, 1140868226, 1140868282, 1140872040, 1140882108, 1140882190, 1140882192, 1140882268, 1140882392, 1140911760, 1141163138, 1141164044, 1141167844, 1140871310, 1140871374, 1140871388, 1140871394, 1140875540, 1140875616, 1140877962, 1140877964, 1140877966, 1140878030, 1140910496, 1140911086, 1140911748, 1140911750, 1140911762, 1140927152, 1140928656, 1141149110, 1141152166, 1141152168, 1141153082, 1141153134, 1141157412, 1141164254, 1141176278, 1141177836, 1141182814, 1141182868, 1141184226, 1141184546, 1141188652, 1141190952, 1141191742, 1141194296, 1141200576, 1141200748, 1140871462, 1140871472, 1140881612, 1140871168, 1140871174, 1140877892, 1140878034, 1140878036, 1140884488, 1140917394, 1140921828, 1141174424, 1141176878, 1141182674, 1141191028, 1141176662, 1141176668, 1141176670, 1140871542, 1140871546, 1141180140, 1141180148, 1141180150, 1141180152, 1140871336, 1141157452
Calcium supplement	6179, 6155		6179 = 3; 6155 = 7
Fish oil supplement or eat oily fish	6179, 1329		6179 = 1, 1329 = 3, 4, 5
Vitamin D supplement	6155		6155 = 4
Hormone replacement therapy	2814	3536, 3546	
Menopause	2724		For 2724 = 2 or 3, menopausal status was adjudicated using hormone replacement therapy status (menopause = yes if hormone replacement therapy = yes) and age at baseline assessment (premenopausal if aged <51 years and menopausal if aged ≥51 years)
Physical activity	864, 874, 884, 894, 904, 914		These fields were used to calculate a summary physical activity measure using the short format calculation of the metabolic equivalent of task in Craig et al[34] and dividing by 1000
Alcohol	1558		

Variable	Data fields	Related age or date fields	Note
Smoking	20116	2879	
Processed meat intake	1349		
Beef intake	1369		
Pork intake	1389		
Cereal intake	1458		
White bread intake	1438, 1448		1448 = 1
Wholemeal/wholegrain bread intake	1438, 1448		1448 = 3
Cooked vegetable intake	1289		
Raw vegetable or salad intake	1299		
Fresh fruit intake	1309		
Dried fruit intake	1319		

Note: * represents a wildcard.
