# Supplementary material for: Colorectal cancer risk prediction using a simple multivariable model
Source: PLoS One. 2025 May 13;20(5):e0321641. doi: 10.1371/journal.pone.0321641 (PMC12074527; doi:10.1371/journal.pone.0321641)
Supplement: S2 Table — (PDF) [file pone.0321641.s004.pdf]

S2 Table. Summary statistics for unaffected and affected women and men for baseline risk factors considered in the development of the colorectal cancer risk prediction models

	Women				Men			
**Risk factor**	**Unaffected**		**Affected**		**Unaffected**		**Affected**	
**Continuous**	**Mean**	**SD**	**Mean**	**SD**	**Mean**	**SD**	**Mean**	**SD**
140-SNP PRS	8.04	0.46	8.24	0.46	8.04	0.46	8.23	0.46
Body mass index (kg/m^2)	27.01	5.15	27.19	5.01	27.83	4.24	28.44	4.31
Physical activity (MET-minutes per week)	2562.89	2494.75	2502.39	2398.12	2853.20	2980.40	2746.06	2899.21
Time since last screening procedure, if screened in last 10 years (years)	4.46	2.94	4.65	3.04	4.38	2.93	4.85	2.87
Cholesterol (mmol/L)	5.90	1.12	6.07	1.16	5.51	1.12	5.40	1.17
High-density lipoprotein (mmol/L)	1.60	0.38	1.60	0.38	1.28	0.31	1.29	0.33
Low-density lipoprotein (mmol/L)	3.64	0.87	3.76	0.89	3.49	0.86	3.40	0.89
Triglycerides (mmol/L)	1.55	0.85	1.70	0.90	1.98	1.15	2.00	1.13
Cooked vegetables (serves per day)	2.65	1.47	2.70	1.45	2.65	1.64	2.73	1.63
Salad or raw vegetables (serves per day)	2.28	1.81	2.28	1.78	1.83	1.73	1.79	1.67
Fresh fruit (pieces per day)	2.35	1.46	2.40	1.44	1.98	1.48	1.98	1.52
**Categorical**	**N**	**%**	**N**	**%**	**N**	**%**	**N**	**%**
Affected first-degree relative, any								
No	188,683	88.9	1,619	84.6	157,154	87.7	2,140	82.4
Yes	22,616	10.7	280	14.6	19,843	11.1	415	16.0
Unknown*	971	0.5	14	0.7	2,294	1.3	43	1.7
Screening procedure in last 10 years								
No	195,012	91.9	1,806	94.4	167,627	93.5	2,474	95.2
Yes	17,258	8.1	107	5.6	11,664	6.5	124	4.8

	Women				Men			
**Risk factor**	**Unaffected**		**Affected**		**Unaffected**		**Affected**	
Diabetes, type 2 or unspecified								
No	205,639	96.9	1,833	95.8	167,956	93.7	2,348	90.4
Yes	6,631	3.1	80	4.2	11,335	6.3	250	9.6
NSAID, regular use								
No	148,835	70.1	1,363	71.3	121,059	67.5	1,664	64.1
Yes	61,541	29.0	525	27.4	56,215	31.4	890	34.3
Unknown	1,894	0.9	25	1.3	2,017	1.1	44	1.7
Menopause and HRT								
Premenopausal	52,229	24.6	196	10.3				
Menopausal, no HRT	76,941	36.3	810	42.3				
Menopausal, took HRT	82,548	38.9	896	46.8				
Missing	552	0.3	11	0.6				
Calcium supplement								
No	148,687	70.1	1,318	68.9	144,255	80.5	2,110	81.2
Yes	63,073	29.7	585	30.6	34,445	19.2	482	18.6
Unknown	510	0.2	10	0.5	591	0.3	3	0.2
Vitamin D supplement								
No	153,065	72.1	1,354	70.8	142,890	79.7	2,091	80.5
Yes	58,364	27.5	547	28.6	35,144	19.6	480	18.5
Unknown	841	0.4	12	0.6	1,257	0.7	27	1.0
Fish oil supplement or eat oily fish two or more times per week								
No	142,033	66.9	1,197	62.6	124,754	69.6	1,741	67.0
Yes	69,629	32.8	705	36.9	53,984	30.1	849	32.7
Unknown	608	0.3	11	0.6	643	0.4	8	0.3

	Women				Men			
**Risk factor**	**Unaffected**		**Affected**		**Unaffected**		**Affected**	
Alcohol use								
Never or rarely	73,547	34.7	689	36.0	36,328	20.3	453	17.4
One or two times per week	56,100	26.4	468	24.5	47,011	26.2	602	23.2
Three of four times per week	46,153	21.7	366	19.1	48,650	27.1	711	27.4
Daily or almost daily	36,221	17.1	384	20.1	47,054	26.2	829	31.9
Unknown	249	0.1	6	0.3	248	0.1	3	0.1
Smoking, ever								
No	125,045	58.9	1,023	53.5	87,901	49.0	1,001	38.5
Yes	86,392	40.7	881	46.1	90,666	50.6	1,588	61.1
Unknown	833	0.4	9	0.5	724	0.4	9	0.4
Processed meat (serves per week)								
None	24,621	11.6	193	10.1	8,376	4.7	80	3.1
1	80,587	38.0	739	38.6	37,135	20.7	518	19.9
2	62,239	29.3	573	30.0	54,294	30.3	803	30.9
3 or more	44,429	20.9	404	21.1	79,109	44.1	1,192	45.9
Unknown	394	0.2	4	0.2	377	0.2	5	0.2
Beef (serves per week)								
None	26,015	12.3	211	11.0	12,028	6.7	127	4.9
1	98,906	46.6	888	46.4	81,239	45.3	1,172	45.1
2	64,067	30.2	587	30.7	62,786	35.2	880	33.9
3 or more	22,479	10.6	218	11.4	22,485	12.5	410	15.8
Unknown	785	0.4	9	0.5	753	0.4	9	0.4

	Women				Men			
**Risk factor**	**Unaffected**		**Affected**		**Unaffected**		**Affected**	
Pork (serves per week)								
None	39,277	18.5	325	17.0	20,675	11.5	267	10.3
1	122,982	57.9	1,104	57.7	105,111	58.6	1,458	56.1
2	43,753	20.6	420	22.0	44,733	25.0	719	27.7
3 or more	5,150	2.4	50	2.6	7,663	4.3	137	5.3
Unknown	1,108	0.5	14	0.7	1,139	0.6	17	0.7
Dried fruit (serves per day)								
None	119,756	56.4	1,071	56.0	122,036	68.1	1,831	70.5
1 or more	90,476	42.6	822	43.0	55,398	30.9	733	28.2
Unknown	2,038	1.0	20	1.1	1,857	1.0	34	1.3
Cereal (bowls per week)								
None	33,790	15.9	320	16.7	30,595	17.1	517	19.9
1–3	35,018	16.5	296	15.5	31,968	17.8	468	18.0
4–6	57,936	27.3	485	25.4	47,219	26.3	629	24.2
7 or more	84,998	40.0	808	42.2	69,000	38.5	977	37.6
Unknown	528	0.3	4	0.2	509	0.3	7	0.3
White bread (slices per week)								
None	170,436	80.3	1,524	79.7	121,247	67.6	1,694	65.2
1–4	6,808	3.2	52	2.7	3,301	1.8	42	1.6
5–10	16,629	7.8	141	7.4	16,884	9.4	253	9.7
11 or more	17,313	8.2	181	9.5	36,586	20.4	580	22.3
Unknown	1,084	0.5	15	0.8	1,273	0.7	29	1.1

	Women				Men			
**Risk factor**	**Unaffected**		**Affected**		**Unaffected**		**Affected**	
Wholemeal or wholegrain bread (slices per week)								
None	83,003	39.1	778	40.7	88,311	49.3	1,326	51.0
1–4	24,257	11.4	188	9.8	6,001	3.4	88	3.4
5–10	53,693	25.3	482	25.2	27,874	15.6	415	16.0
11 or more	50,042	23.6	447	23.4	56,168	31.3	746	28.7
Unknown	1,275	0.6	18	0.9	937	0.5	23	0.9

Note: HRT, hormone replacement therapy; MET, metabolic equivalent task; NSAID, non-steroidal anti-inflammatory drug; PRS, polygenic risk score; SD, standard deviation; SNP, single-nucleotide polymorphism.

Body mass index was missing for 625 (0.3%) unaffected and 5 (0.3%) affected women and for 636 (0.4%) unaffected and 9 (0.3%) affected men; physical activity was missing for 53,582 (25.2%) unaffected and 532 (27.8%) affected women and for 31,500 (17.6%) unaffected and 505 (19.4%) affected men; total cholesterol was missing for 9,999 (4.7%) unaffected and 90 (4.7%) affected women and for 8,192 (4.6%) unaffected and 137 (5.3%) affected men; high-density lipoprotein was missing for 28,497 (13.4%) unaffected and 246 (12.9%) affected women and for 21,407 (11.9%) unaffected and 323 (12.4%) affected men; low-density lipoprotein was missing for 10,338 (4.9%) unaffected and 92 (4.8%) affected women and for 8,561 (4.8%) unaffected and 141 (5.4%) affected men; triglycerides was missing for 10,113 (4.8%) unaffected and 89 (4.7%) affected women and for 8,370 (4.7%) unaffected and 142 (5.5%) affected men; cooked vegetables consumption was missing for 1,675 (0.8%) unaffected and 17 (0.9%) affected women and for 2,650 (1.5%) unaffected and 45 (1.7%) affected men; salad or raw vegetables consumption was missing for 2,016 (0.9%) unaffected and 21 (1.1%) affected women and for 2,861 (1.6%) unaffected and 45 (1.7%) affected men; fresh fruit consumption was missing for 676 (0.3%) unaffected and 5 (0.3%) affected women and for 891 (0.5%) unaffected and 20 (0.8%) affected men; other continuous variables had no missing data.

* Unknown is no response to family history questions for all of mother, father and siblings. A further 1,628 (0.8%) unaffected and 18 (0.9%) affected women and 2,775 (1.5%) unaffected and 49 (1.9%) affected men were missing for mother and father but not for siblings; 839 (0.4%) unaffected and 5 (0.3%) affected women and 1,769 (1.0%) unaffected and 33 (1.3%) affected men were missing for mother and siblings but not for father; 1,417 (0.7%) unaffected and 14 (0.7%) affected women and 1,893 (1.1%) unaffected and 25 (9.6%) affected men were missing for father and siblings but not for mother; 3,591 (1.7%) unaffected and 29 (1.5%) affected women and 4,716 (2.6%) unaffected and 68 (2.6%) affected men were missing mother only; 10,655 (5.0%) affected and 102 (5.3%) affected women and 8,834 (4.9%) affected and 150 (5.8%) affected men were missing father only; 6,098 (2.9%) affected were and 65 (3.4%) unaffected women and 7,503 (4.2%) affected were and 137 (5.3%) unaffected men were missing sibling only.
