# Supplementary material for: Colorectal cancer risk prediction using a simple multivariable model
Source: PLoS One. 2025 May 13;20(5):e0321641. doi: 10.1371/journal.pone.0321641 (PMC12074527; doi:10.1371/journal.pone.0321641)
Supplement: S3 Table — (PDF) [file pone.0321641.s005.pdf]

S3 Table. Unadjusted hazard ratios for women and men for the baseline risk factors considered in the development of the colorectal cancer risk prediction models using the multiple imputation data for the 70% training dataset

	Women			Men		
**Risk factor**	**Hazard ratio**	**95% confidence interval**	**P value**	**Hazard ratio**	**95% confidence interval**	**P value**
**Continuous**						
140-SNP PRS (standardised)	1.519	1.438, 1.605	<0.001	1.500	1.431, 1.572	<0.001
Body mass index (natural log of kg/m^2 centred)	1.067	0.786, 1.449	0.7	2.648	1.937, 3.620	<0.001
Time since last screening procedure, if screened in last 10 years (years)	1.019	0.945, 1.100	0.6	1.090	1.016, 1.169	0.02
Physical activity (natural log of MET-minutes per week, centred)	0.978	0.940, 1.018	0.3	0.978	0.947, 1.010	0.2
Cholesterol (mmol/L, centred)	1.056	1.007, 1.107	0.03	0.977	0.938, 1.019	0.3
High-density lipoprotein (mmol/L, centred)	0.945	0.815, 1.095	0.5	0.906	0.779, 1.054	0.2
Low-density lipoprotein (mmol/L, centred)	1.069	1.005, 1.137	0.03	0.963	0.912, 1.017	0.2
Triglycerides (mmol/L, centred)	1.096	1.032, 1.164	0.003	1.058	1.016, 1.101	0.006
Cooked vegetables (serves per day)	1.003	0.966, 1.040	0.9	1.007	0.979, 1.036	0.6
Salad or raw vegetables (serves per day)	1.010	0.980, 1.040	0.5	0.979	0.953, 1.007	0.1
Fresh fruit (pieces per day)	0.992	0.956, 1.030	0.7	0.993	0.962, 1.024	0.6
**Categorical**						
Affected first-degree relative, any						
No	–			–		
Yes	1.286	1.102, 1.499	0.001	1.439	1.271, 1.630	<0.001
Screening procedure in last 10 years						
No	–			–		
Yes	0.617	0.490, 0.778	<0.001	0.683	0.552, 0.845	<0.001

	Women			Men		
**Risk factor**	**Hazard ratio**	**95% confidence interval**	**P value**	**Hazard ratio**	**95% confidence interval**	**P value**
Diabetes, type 2 or unspecified						
No	–			–		
Yes	1.070	0.811, 1.412	0.6	1.323	1.132, 1.547	<0.001
NSAID, regular use						
No	–			–		
Yes	0.984	0.874, 1.108	0.8	0.994	0.901, 1.096	0.9
Menopause and HRT (women only)						
Premenopausal	–					
Menopausal, no HRT	1.306	1.006, 1.696	0.05			
Menopausal, took HRT	1.226	0.941, 1.598	0.1			
Calcium supplement						
No	–			–		
Yes	1.017	0.905, 1.142	0.8	0.952	0.846, 1.071	0.4
Vitamin D supplement						
No	–					
Yes	1.043	0.926, 1.174	0.5	0.929	0.825, 1.045	0.2
Fish oil supplement or eat oily fish two or more times per week						
No	–			–		
Yes	0.991	0.888, 1.105	0.9	0.944	0.860, 1.036	0.2
Alcohol use						
Never or rarely	–					
One or two times per week	0.958	0.832, 1.102	0.5	1.091	0.943, 1.261	0.2
Three of four times per week	0.900	0.773, 1.047	0.2	1.146	0.995, 1.321	0.06
Daily or almost daily	1.112	0.958, 1.291	0.2	1.300	1.133, 1.492	<0.001

	Women			Men		
**Risk factor**	**Hazard ratio**	**95% confidence interval**	**P value**	**Hazard ratio**	**95% confidence interval**	**P value**
Smoking, ever						
No	–					
Yes	1.240	1.114, 1.382	<0.001	1.388	1.261, 1.527	<0.001
Processed meat (serves per week)						
None	–			–		
1	1.055	0.874, 1.273	0.6	1.200	0.913, 1.577	0.2
2	1.135	0.936, 1.376	0.2	1.324	1.015, 1.728	0.04
3 or more	1.133	0.925, 1.387	0.2	1.420	1.092, 1.845	0.009
Beef (serves per week)						
None	–			–		
1	1.101	0.917, 1.322	0.3	1.219	0.981, 1.514	0.07
2	1.103	0.910, 1.335	0.3	1.180	0.947, 1.470	0.1
3 or more	1.054	0.836, 1.329	0.7	1.540	1.218, 1.948	<0.001
Pork (serves per week)						
None	–			–		
1	1.045	0.900, 1.213	0.6	1.019	0.871, 1.191	0.8
2	1.082	0.910, 1.287	0.9	1.186	1.003, 1.402	0.05
3 or more	1.121	0.785, 1.601	0.6	1.432	1.118, 1.833	0.004
Dried fruit (serves per day)						
None	–			–		
1 or more	0.974	0.873, 1.085	0.6	0.849	0.767, 0.940	0.002

	Women			Men		
Risk factor	Hazard ratio	95% confidence interval	P value	Hazard ratio	95% confidence interval	P value
Cereal (bowls per week)						
None	–			–		
1–3	0.891	0.736, 1.079	0.2	0.901	0.776, 1.045	0.2
4–6	0.917	0.775, 1.086	0.3	0.740	0.643, 0.851	<0.001
7 or more	0.884	0.756, 1.033	0.1	0.721	0.635, 0.819	<0.001
White bread (slices per week)						
None	–			–		
1–4	1.007	0.726, 1.398	1.0	0.984	0.681, 1.420	0.9
5–10	1.005	0.816, 1.238	1.0	1.103	0.940, 1.295	0.2
11 or more	1.166	0.969, 1.403	0.1	1.180	1.054, 1.320	0.004
Wholemeal or wholegrain bread (slices per week)						
None	–			–		
1–4	0.839	0.695, 1.014	0.07	0.953	0.730, 1.243	0.7
5–10	0.917	0.801, 1.050	0.2	0.990	0.868, 1.129	0.9
11 or more	0.863	0.751, 0.992	0.04	0.901	0.810, 1.002	0.06

Note: HRT, hormone replacement therapy; MET, metabolic equivalent task; NSAID, non-steroidal anti-inflammatory drug; PRS, polygenic risk score; SNP, single-nucleotide polymorphism.
