# Supplementary material for: Colorectal cancer risk prediction using a simple multivariable model
Source: PLoS One. 2025 May 13;20(5):e0321641. doi: 10.1371/journal.pone.0321641 (PMC12074527; doi:10.1371/journal.pone.0321641)
Supplement: S4 Table — (PDF) [file pone.0321641.s006.pdf]

S4 Table. Nelson–Aalen cumulative hazards extracted at 5-year intervals from age 40 to 80 years separately for women and men for the risk prediction models.

Women					Men				
Age (years)	Number at risk	Number affected	Cumulative hazard	95% CI	Age (years)	Number at risk	Number affected	Cumulative hazard	95% CI
**Average risks**					**Average risks**				
Quintile 1					***Quintile 1***				
55	7,026	6	0.004	0.002, 0.005	55	5,724	10	0.003	0.002, 0.005
65	0	0			65	0	0		
75	0	0			75	0	0		
Quintile 2					***Quintile 2***				
55	11,316	39	0.002	0.001, 0.003	55	8,822	40	0.005	0.003, 0.010
65	1,026	0	0.011	0.008, 0.014	65	1,599	2	0.017	0.013, 0.022
75	0	0			75	0	0		
Quintile 3					***Quintile 3***				
55	0	14	0.000		55	0	12	0.000	
65	13,043	32	0.007	0.005, 0.009	65	11,157	59	0.011	0.008, 0.015
75	0	0			75	0	0		
Quintile 4					***Quintile 4***				
55	0	0	0.000		55	0	0	0.000	
65	12,715	71	0.005	0.003, 0.007	65	10,194	92	0.005	0.003, 0.008
75	64	0	0.016	0.013, 0.020	75	315	0	0.028	0.023, 0.034
Quintile 5					***Quintile 5***				
55	0	0	0.000		55	0	0	0.000	
65	990	45	0.003	0.000, 0.019	65	0	54	0.000	
75	10,762	35	0.017	0.012, 0.024	75	10,206	48	0.022	0.018, 0.026

Women					Men				
Age (years)	Number at risk	Number affected	Cumulative hazard	95% CI	Age (years)	Number at risk	Number affected	Cumulative hazard	95% CI
**Family history alone model**					**Family history alone model**				
Quintile 1					***Quintile 1***				
55	7,172	10	0.003	0.002, 0.004	55	5,832	11	0.004	0.003, 0.005
65	0	0			65	0	0		
75	0	0			75	0	0		
Quintile 2					***Quintile 2***				
55	9,861	36	0.008	0.004, 0.019	55	7,868	35	0.004	0.003, 0.008
65	2,335	0	0.016	0.011, 0.025	65	2,472	6	0.016	0.012, 0.020
75	0	0			75	0	0		
Quintile 3					***Quintile 3***				
55	718	10	0.001	0.000, 0.010	55	566	14	0.000	
65	11,823	32	0.009	0.006, 0.014	65	10,770	62	0.014	0.010, 0.019
75	0	0			75	0	0		
Quintile 4					***Quintile 4***				
55	510	2	0.000		55	280	1	0.000	
65	10,391	76	0.009	0.005, 0.019	65	7,189	77	0.014	0.008, 0.026
75	2,077	1	0.020	0.014, 0.029	75	2,491	0	0.034	0.026, 0.045
Quintile 5					***Quintile 5***				
55	81	1	0.000		55	0	1	0.000	
65	3,225	40	0.007	0.004, 0.014	65	2,519	62	0.008	0.004, 0.015
75	8,749	34	0.023	0.018, 0.029	75	8,030	48	0.033	0.027, 0.040

Women					Men				
Age (years)	Number at risk	Number affected	Cumulative hazard	95% CI	Age (years)	Number at risk	Number affected	Cumulative hazard	95% CI
**Current family history and PRS model**					**Current family history and PRS model**				
Quintile 1					***Quintile 1***				
55	6,819	7	0.002	0.001, 0.004	55	5,639	10	0.003	0.002, 0.005
65	431	0	0.004	0.003, 0.007	65	157	0	0.007	0.005, 0.011
75	4	0	0.004	0.003, 0.007	75	1	0	0.007	0.005, 0.011
Quintile 2					***Quintile 2***				
55	7,234	17	0.005	0.003, 0.009	55	6,345	19	0.005	0.003, 0.008
65	4,554	8	0.010	0.007, 0.014	65	3,787	10	0.011	0.008, 0.015
75	311	0	0.015	0.011, 0.021	75	254	0	0.020	0.014, 0.028
Quintile 3					***Quintile 3***				
55	2,847	21	0.005	0.002, 0.010	55	1,817	19	0.022	0.006, 0.083
65	8,078	25	0.014	0.010, 0.019	65	7,108	32	0.036	0.016, 0.081
75	1,574	2	0.023	0.018, 0.029	75	1,729	4	0.048	0.026, 0.089
Quintile 4					***Quintile 4***				
55	1,047	9	0.027	0.005, 0.144	55	534	10	0.000	
65	8,049	32	0.035	0.010, 0.126	65	6,670	66	0.014	0.010, 0.020
75	3,469	14	0.045	0.016, 0.122	75	3,440	11	0.033	0.027, 0.039
Quintile 5					***Quintile 5***				
55	395	5	0.000		55	211	4	0.000	
65	6,662	83	0.014	0.009, 0.021	65	5,228	99	0.018	0.013, 0.025
75	5,468	19	0.033	0.027, 0.041	75	5,097	33	0.048	0.041, 0.056

Women					Men				
Age (years)	Number at risk	Number affected	Cumulative hazard	95% CI	Age (years)	Number at risk	Number affected	Cumulative hazard	95% CI
**New family history and PRS model**					**New family history and PRS model**				
Quintile 1					***Quintile 1***				
55	6,739	6	0.003	0.002, 0.004	55	5,520	8	0.004	0.002, 0.005
65	255	0	0.005	0.003, 0.007	65	64	0	0.007	0.005, 0.011
75	3	0	0.005	0.003, 0.007	75	0	0		
Quintile 2					***Quintile 2***				
55	8,089	16	0.004	0.002, 0.008	55	7,152	27	0.007	0.004, 0.013
65	4,013	4	0.009	0.006, 0.012	65	3,301	5	0.014	0.01, 0.02
75	171	0	0.012	0.009, 0.018	75	126	1	0.024	0.015, 0.038
Quintile 3					***Quintile 3***				
55	2,782	26	0.022	0.004, 0.122	55	1,581	17	0.002	0.001, 0.007
65	8,543	23	0.032	0.010, 0.104	65	7,686	34	0.015	0.011, 0.020
75	1,239	1	0.040	0.016, 0.103	75	1,401	3	0.027	0.022, 0.033
Quintile 4					***Quintile 4***				
55	641	9	0.003	0.000, 0.020	55	252	9	0.000	
65	8,664	42	0.012	0.007, 0.020	65	7,073	64	0.015	0.011, 0.021
75	3,289	9	0.022	0.016, 0.030	75	3,338	7	0.032	0.026, 0.038
Quintile 5					***Quintile 5***				
55	91	2	0.000		55	41	1	0.000	
65	6,299	79	0.016	0.009, 0.029	65	4,826	104	0.017	0.011, 0.025
75	6,124	25	0.035	0.026, 0.046	75	5,656	37	0.047	0.040, 0.056

	Women					Men			
Age (years)	Number at risk	Number affected	Cumulative hazard	95% CI	Age (years)	Number at risk	Number affected	Cumulative hazard	95% CI
**New multivariable model**					**New multivariable model**				
Quintile 1					***Quintile 1***				
55	6,697	7	0.002	0.001, 0.004	55	5,549	9	0.003	0.002, 0.005
65	396	0	0.004	0.003, 0.006	65	139	0	0.009	0.005, 0.016
75	10	0	0.004	0.003, 0.006	75	2	0	0.009	0.005, 0.016
Quintile 2					***Quintile 2***				
55	7,735	14	0.005	0.002, 0.008	55	6,762	23	0.013	0.005, 0.037
65	4,131	6	0.008	0.005, 0.012	65	3,495	6	0.019	0.009, 0.039
75	296	0	0.017	0.012, 0.025	75	221	0	0.025	0.014, 0.045
Quintile 3					***Quintile 3***				
55	3,037	24	0.012	0.002, 0.062	55	1,810	21	0.004	0.002, 0.010
65	8,152	18	0.023	0.010, 0.055	65	7,312	31	0.018	0.014, 0.024
75	1,381	1	0.028	0.014, 0.058	75	1,542	4	0.030	0.024, 0.036
Quintile 4					***Quintile 4***				
55	739	12	0.002	0.000, 0.016	55	372	7	0.003	0.001, 0.023
65	8,625	41	0.013	0.008, 0.020	65	6,953	67	0.014	0.008, 0.024
75	3,209	9	0.024	0.019, 0.032	75	3,345	11	0.032	0.024, 0.041
Quintile 5					***Quintile 5***				
55	134	2	0.000		55	53	2	0.000	
65	6,470	83	0.011	0.008, 0.017	65	5,051	103	0.023	0.015, 0.035
75	5,930	25	0.030	0.025, 0.036	75	5,411	33	0.054	0.045, 0.065

Note: CI, confidence interval; PRS, polygenic risk score
